# Aquaporin water channels: distribution, physiology, pathophysiology, and relevance as therapeutic targets

**DOI:** 10.1186/s43556-026-00628-9

**Published:** 2026-10-25

**Authors:** Lin Wang, Yanqun Luo, Xiaoliang Deng, Junming Chen, Lei Wang, Tao Wu

**Affiliations:** 1 Department of Hepatology, Longhua Hospital, Shanghai University of Traditional Chinese Medicine, Shanghai, 200032 China; 2 Institute of Interdisciplinary Integrative Medicine Research, Shanghai University of Traditional Chinese Medicine, Shanghai, 201203 China; 3 Minhang Hospital, Fudan University, Shanghai, 201199 China

**Keywords:** Aquaporins, Physiological functions, Pathological mechanisms, Disease targets, Therapeutic strategies

## Abstract

Aquaporins (AQPs) represent a class of integral membrane proteins that function as selective water channels, facilitating the transit of water molecules across biological membranes. Under specific conditions, these proteins may also mediate the permeation of small solutes, including glycerol, hydrogen peroxide, urea, carbon dioxide, and ammonia. Investigations into AQPs have unveiled distinctive structural attributes and functional properties. Evidence indicates that AQPs are broadly distributed across diverse tissues and organ systems in humans, contributing to essential physiological processes. An expanding body of literature highlights the involvement of AQPs in disease pathogenesis and progression, particularly in cellular behaviors such as proliferation and motility, pulmonary gas exchange, and the modulation of non-canonical signaling pathways. This review comprehensively outlines the tissue-specific and subcellular distribution profiles, physiological roles, and regulatory mechanisms associated with AQPs. It further characterizes the alterations in AQP functionality under pathological states, delineates their involvement and mechanistic contributions to multisystem disorders, and provides an overview of therapeutic strategies targeting AQPs, along with their translational prospects. In addition, key gaps in current knowledge are identified, and pivotal research directions, such as tissue-specific modulation and the design of targeted inhibitors and activators, are proposed to advance the translational application of AQPs as diagnostic and therapeutic targets.

## Introduction

Aquaporins (AQPs) are integral membrane channels that form tetramers in biological membranes, while each monomer contains an independent permeation pathway. The first identified member was the 28-kDa channel-forming integral membrane protein CHIP28, later renamed AQP1 after its water-channel activity was confirmed [1, 2]. Thirteen AQP isoforms have been identified in humans. Depending on the isoform and experimental conditions, AQPs can also facilitate the transport of glycerol, hydrogen peroxide (H_2_O_2_), ammonia, urea, gases, and, in a few cases, ions [3, 4]. These differences, together with tissue distribution, membrane polarity, organellar localization, and interacting proteins, underline their functional specialization.

Human AQPs are generally divided into three evolutionary subfamilies: orthodox aquaporins, including AQP0, AQP1, AQP2, AQP4, AQP5, AQP6, and AQP8; aquaglyceroporins, including AQP3, AQP7, AQP9, and AQP10; and non-orthodox aquaporins, represented by AQP11 and AQP12 [5]. Orthodox AQPs are primarily water-selective channels; however, AQP6 exhibits pH-dependent anion conductance under acidic conditions, whereas AQP8 facilitates the transport of neutral ammonia and H_2_O_2_ [6–8]. Aquaglyceroporins conduct water and glycerol and may also transport other small neutral solutes. AQP11 and AQP12 are phylogenetically divergent, predominantly intracellular aquaporins. AQP11 mainly resides in the endoplasmic reticulum membrane in several tissues, whereas AQP12 is enriched in pancreatic acinar β cells and can traffic from the endoplasmic reticulum to zymogen granules following secretagogue stimulation. AQP tissue and subcellular distributions are therefore highly isoform-specific [9, 10]. AQP2 is enriched in renal collecting duct principal cells [11], AQP4 is polarized to perivascular astrocytic end-feet [12], and AQP5 is expressed in secretory epithelia [13] and pulmonary alveolar epithelial cells [14]. AQP7 is abundant in adipocytes [15] whereas human AQP9 is prominent in hepatocytes [16] and peripheral leukocytes [17]. AQP12 is enriched in pancreatic acinar cells [9]. These distribution patterns are dynamically regulated by hormonal stimulation, developmental and metabolic states, aging, and disease-associated trafficking or loss of polarity.

The physiological functions of AQPs extend from fluid homeostasis and epithelial secretion to energy metabolism, redox regulation, edema dynamics, and tissue repair. AQP1 and AQP2 [11, 18] support renal water reabsorption, AQP4 [19] regulates brain water movement and glymphatic transport, and AQP5 [20] contributes to salivary, lacrimal, and airway fluid secretion. Aquaglyceroporins coordinate adipose glycerol release and hepatic glycerol uptake, whereas several AQP isoforms facilitate H_2_O_2_ transport and redox-sensitive signaling [21, 22]. AQPs also regulate cell proliferation, migration, apoptosis, inflammation, and angiogenesis. These effects can involve substrate transport, including water, glycerol, and H_2_O_2_, but may also be coupled to PI3K/Akt, MAPK, NF-κB, Wnt/β-catenin, cytoskeletal, and vesicular-trafficking networks [23, 24]. The pathological effects of AQPs are strongly dependent on isoform, tissue, subcellular localization, disease stage, and cellular background. AQP2 mutations or defective trafficking cause nephrogenic diabetes insipidus [25, 26], AQP4 exerts edema type- and stage-dependent effects in the injured brain [27], and AQP11 dysfunction disturbs polycystin-1 processing and promotes renal cystogenesis [28]. AQP7 deficiency causes adipose tissue remodeling, inflammation, and metabolic dysregulation [15]. In cancer, different AQPs are linked to distinct phenotypes: AQP1 mainly supports angiogenesis, AQP3 facilitates H_2_O_2_-dependent migration and invasion [23], AQP5 promotes epithelial–mesenchymal transition and therapy resistance [24, 29], and AQP8 shows tumor-type-dependent effects [30, 31] shows tumor-type-dependent effects. Therefore, measuring total AQP expression alone is insufficient to determine whether a particular isoform is protective, pathogenic, or simply a correlate of the disease state.

AQP-directed therapy remains attractive because these proteins reside at defined membrane and organellar sites and participate in several disease-relevant processes [32]. However, the available evidence remains difficult to translate into clinical practice. Many reported inhibitors have limited affinity or isoform selectivity, while direct pore blockade is complicated by the narrow and highly conserved channel architecture [33, 34]. Experimental conclusions are also confounded by species differences, functional redundancy among AQP isoforms, off-target effects of compounds, antibody specificity, and reliance on expression data without direct measurement of membrane localization or substrate permeability [35]. A more useful evaluation should separate correlation from causation and consider isoform, cell type, subcellular compartment, disease phase, and transported substrate [35, 36]. Given these challenges, this review examines the 13 human AQPs through the linked framework of subfamily classification, substrate selectivity, tissue and subcellular distribution, physiological function, molecular dysfunction, disease involvement, and therapeutic targeting [5]. Particular attention is paid to contradictory findings, human–animal differences, and the experimental evidence required before AQPs can be considered viable diagnostic or therapeutic targets.

## AQP subfamilies, substrate selectivity, and context-dependent localization

Human AQPs are grouped by primary-sequence relationships into three evolutionary subfamilies: orthodox aquaporins (AQP0, AQP1, AQP2, AQP4, AQP5, AQP6, and AQP8), aquaglyceroporins (AQP3, AQP7, AQP9, and AQP10), and non-orthodox aquaporins (AQP11 and AQP12) [37]. This classification provides a useful starting point, but it does not fully predict substrate selectivity or in vivo function. AQP expression is highly dependent on cell type, subcellular compartment, and species, and broad organ-level distribution maps can obscure clinically important differences in membrane polarity, organellar localization, and disease-stage-dependent redistribution. We therefore summarize each subfamily below, and in the subsequent disease-based sections we evaluate the functional significance of individual isoforms.

### Orthodox aquaporins

Orthodox aquaporins are generally optimized for water transport, but “water-selective” should not be interpreted as universally water-exclusive. AQP6 can function as an anion channel under acidic conditions. The substrate permeability of AQP8 is species- and assay-dependent. Human AQP8 facilitates the transport of water, ammonia, and hydrogen peroxide but does not show detectable permeability to urea or glycerol. By contrast, urea permeability has been reported for mouse AQP8 in heterologous expression systems. AQP8 is mainly expressed in hepatocytes, although its subcellular distribution and transport properties vary across species and experimental models [38, 39]. At the cellular level, orthodox aquaporins are chiefly localized within specialized epithelial and endothelial cells across diverse tissues, where they function as primary mediators of transcellular water movement.

AQP0, originally identified as the major intrinsic protein (MIP) of the lens, is predominantly expressed in lens fiber cells, where its expression level is closely linked to lens transparency. Mutations in AQP0 cause congenital cataracts in both humans and mice [40]. AQP0 is also present in early erythroid cells, particularly during the R1 and R2 phases. AQP0 expression can serve as an indicator of dyserythropoiesis [41]. AQP1 is one of the most widely expressed aquaporins in mammalian tissues. In human and mouse kidneys, AQP1 is localized in the terminal segment of the renal proximal tubule, the thin descending limb of the loop of Henle, and the endothelial lining of the descending vasa recta. AQP1 is also present in the ocular lens, ciliated epithelium of the ocular surface, myocardium, pulmonary endothelial cells, fibroblasts, mesothelial layers, and the choroid plexus ependyma. AQP1 critically participates in renal water reabsorption, aqueous humor secretion, and cerebrospinal fluid production, thereby modulating intraocular and intracranial pressures [42, 43]. AQP1 plays a fundamental role in regulating fluid homeostasis in multiple organ systems.

AQP2 shows a highly tissue-specific expression pattern, predominantly expressed in the kidney, where it resides in the principal cells of the collecting ducts spanning from the cortex to the medulla. AQP2 functions as the central mediator of vasopressin-regulated water reabsorption in the kidney [44]. Alterations in AQP2 expression or its subcellular mislocalization are the major molecular basis of nephrogenic diabetes insipidus. Moreover, quantification of urinary AQP2 serves as a diagnostic biomarker for evaluating the renal responsiveness to antidiuretic hormone [45].

AQP4 is the most abundant water channel in the central nervous system and is primarily localized to astrocyte-blood vessel and astrocyte-cerebrospinal fluid interfaces. Under normal conditions, AQP4 facilitates the bidirectional movement of water to help preserve fluid balance in the brain [46]. In response to injuries such as trauma or stroke, excessive water entry through AQP4 contributes to cytotoxic edema and a rise in intracranial pressure. The presence of AQP4 at the cell membrane is tightly controlled through vesicle trafficking, a process involving dynamin-dependent internalization, Rab5/Rab11-associated endosomes, and cytoskeletal elements. Therefore, current therapeutic interest has shifted from direct channel blockade toward modulating its subcellular localization to manage brain swelling [47]. Astrocytes from both mice and humans display several AQP4 isoforms, which are thought to form the basis of the astrocyte membrane structures referred to as “orthogonal arrays of intramembranous particles” [48].

AQP5 is expressed at the plasma membrane of type I alveolar epithelial cells, as well as in submucosal glands and airway epithelial cells [49]. Owing to its approximately 63% sequence identity with AQP2, it has been inferred that AQP5 may share mechanistic parallels in water transport [50]. Beyond its canonical role in fluid homeostasis, AQP5 has been shown to activate the Ras-MAPK signaling axis in various cancers, an activity implicated in the growth and metastatic dissemination of both lung cancer and glioma cells [51, 52]. In exocrine physiology, AQP5 also has key functions: it resides at the apical membrane of salivary acinar cells and within lacrimal acinar cells, where it facilitates saliva and tear secretion. Notably, in Sjögren's syndrome, reduced AQP5 expression or its mislocalization to aberrant subcellular compartments has been robustly correlated with diminished secretory output from affected glands [53].

AQP6 is localized in intracellular vesicles of acid-secreting intercalated cells in the renal collecting duct, where it functions primarily as an anion channel and co-localizes with H^+^-ATPase [54, 55]. At higher pH, AQP6 exhibits restricted water permeability at the plasma membrane, and its direct involvement in renal pathology remains to be elucidated.

AQP8 can transport small molecules such as ammonia, hydrogen peroxide, and water, and it is primarily expressed in hepatocytes. In the basal state, AQP8 in hepatocytes shows a heterogeneous subcellular distribution, with the majority (75%) residing within intracellular vesicles and the remainder localized on the plasma membrane [56, 57]. Upon cyclic adenosine monophosphate (cAMP) stimulation, AQP8 relocates to the plasma membrane, contributing to the regulation of bile secretion within the bile canaliculi [58, 59].

### Aquaglyceroporins

Aquaglyceroporins facilitate the transmembrane movement of both water and glycerol. Certain isoforms within this group also enable the passage of hydrogen peroxide, urea, and ammonia [60]. These channels are predominantly expressed in metabolic organs such as the liver, adipose tissue, skin, and small intestine [61]. At the cellular level, they localize mainly to metabolic epithelial cells and epidermal cells. By mediating glycerol flux and contributing to energy metabolism and lipid homeostasis, they play an integral role in metabolic regulation and bear pivotal relevance to metabolic disorders [62, 63].

AQP3 is mainly localized to the basal epidermal cells of human skin, where it modulates signal transduction, affects downstream protein expression, and promotes tumorigenesis and cell migration, thereby significantly influencing tumor biology [64]. Research indicates that AQP3 is overexpressed in human squamous cell carcinomas, and mice with a specific deletion of the AQP3 gene exhibit a reduced occurrence of skin tumors [65, 66]. AQP3 facilitates the supply of essential glycerol for lipid structure synthesis and enhances cell proliferation and growth by increasing ATP generation [67]. AQP3 has been implicated in tumorigenesis through multiple molecular mechanisms [68]. Notably, mice lacking AQP3 and exposed to a tumor-inducing agent exhibit suppressed skin cancer development.

AQP7 is predominantly expressed in adipose tissue in humans and rodents, where it facilitates glycerol transport across membranes. Several studies have indicated that both AQP7 expression and its translocation to the plasma membrane are associated with the hydrolysis of triglycerides to glycerol [69]. AQP7 expression is altered in adipose tissue of individuals with obesity [70]. AQP7 expression is markedly upregulated in peripheral blood of individuals with obesity [71], positioning AQP7 as a potential modulator of metabolic disorders.

Brain expression of AQP9 is species dependent. AQP9 is detectable in healthy astrocytes in mice and rats but is not normally detected in healthy human astrocytes. In humans, AQP9 is expressed predominantly in hepatocytes and leukocytes. This stimulation leads to the production of a significant number of inflammatory cytokines with pyrogenic properties by immune cells, triggering an intensified immune reaction. AQP9 is involved in the early phase of the acute response to endotoxin-induced shock via the NF-κB signaling pathway, which is associated with the progression of inflammation and infection [72].

AQP10 is expressed in the human small intestine and adipose tissue, where it can provide an alternative route for glycerol efflux when AQP7 function is limited. Hepatic AQP10 expression is reduced in human obstructive cholestasis [73]. Importantly, *Aqp10* is a pseudogene in mice, precluding direct investigation of human AQP10 biology in conventional murine models [74]. This species difference may help explain why *Aqp7*-null mice develop obesity, whereas humans with loss-of-function AQP7 variants may show little overt metabolic disturbance: human AQP10 can compensate for impaired AQP7-mediated glycerol efflux [75]. The presence of multiple AQP7-related pseudogene sequences in the human genome further illustrates that pseudogenization, paralog retention, and compensatory capacity differ among species. Such differences should be explicitly stated when extrapolating rodent findings to human disease.

### Non-orthodox aquaporins

Non-orthodox AQPs share relatively low sequence homology with other members of the AQP family [76]. Their substrate selectivity remains incompletely characterized. Available evidence suggests that they may transport water and selected small solutes, including hydrogen peroxide, depending on the isoform and cellular context [35, 77]. These proteins show a relatively restricted tissue distribution and are predominantly localized to intracellular organelle membranes, particularly the endoplasmic reticulum, rather than the plasma membrane [78]. Although their physiological functions remain poorly understood, specific subtypes have been implicated in organelle homeostasis and organ-specific diseases, including polycystic kidney disease [79, 80]. AQP11 and AQP12 are the most divergent paralogs within the AQP family and are in intracellular organelle membranes rather than the plasma membrane, which challenges their categorization as conventional AQPs or aquaglyceroporins [37, 81].

AQP11 exhibits high expression in the testes and thymus, with comparatively lower expression in the kidneys, liver, and intestines. Within the kidney, AQP11 is localized to the proximal tubules, and loss of its function may contribute to cyst development. AQP11 plays a role in the glycosylation process and facilitates the transport of polycystin-1 (PC-1) from the endoplasmic reticulum to the plasma membrane. The lack of PC-1 can result in the onset of polycystic kidney disease [82].

Compared with AQP11, AQP12 is less well characterized. AQP12 is predominantly expressed in human pancreatic acinar cells and is likely involved in the secretion of digestive enzymes and fluids [81]. AQP12-knockout mice show normal growth, blood composition and related parameters, pancreatic juice output, and tissue structure and function. In experimentally induced pancreatitis, AQP12-knockout mice showed more severe pathological damage than wild-type (WT) mice, although AQP12 water permeability (1.9 × 10⁻^3^ cm/s) did not differ significantly between groups [83].

In conclusion, orthodox AQPs and aquaglyceroporins are primarily situated on the plasma membrane and are expressed in key organs involved in fluid homeostasis and metabolic regulation [5, 84]. They act as key regulators of systemic fluid and metabolic homeostasis [35]. Disruptions in their tissue- and cell-specific localization can contribute to pathological conditions such as fluid and electrolyte disturbances and metabolic dysregulation [85]. In contrast, non-orthodox AQPs are predominantly localized to organellar membranes and exhibit narrow tissue tropism; their dysfunction is predominantly associated with organ-specific injury, as summarized in Table 1. This distinctive localization not only underpins the functional specialization of AQPs but also establishes a molecular foundation for precise therapeutic targeting in disease intervention [86].

**Table 1:** The human aquaporin family

Member	Type	Site of expression	Function	Reference
AQP0	I	Lens fiber cells; early erythroid cells	Criteria for the diagnosis of dyserythropoiesis, Control the transparency of the lens	[40, 41]
AQP1	I	Kidney, lens, heart, lung	Mediating renal water reabsorption, aqueous humor secretion, and CSF production; regulating intraocular/intracranial pressure; maintains multi-organ fluid homeostasis	[42, 43]
AQP2	I	principal cell of renal collecting duct	Central mediator of vasopressin-regulated water reabsorption in renal collecting duct principal cells; promoting water reabsorption, urine concentration, and body fluid balance	[44, 45]
AQP3	II	Skin, Lung, cornea, stomach, liver, sperm	Regulating cellular signal transduction and downstream protein expression; promoting glycerol transport for lipid synthesis; increasing ATP production to facilitate cell proliferation and growth; promoting cell migration and tumorigenesis	[65–67]
AQP4	I	Epithelial cells of many organs, Central nervous system	The major water channel in the CNS, maintaining brain fluid balance; mediating cytotoxic edema and elevating intracranial pressure upon injury	[47, 48]
AQP5	I	Saliva, tears and lung secretions	Promoting saliva and tear secretion; activating Ras-MAPK to participate in the growth and metastasis of lung cancer and glioma; reducing or mislocalizing expression in Sjögren's syndrome leads to decreased secretion	[51–53]
AQP6	I	kidney, Retina, ovary, parotid gland, and cochlear sensory epithelium	AQP6 acts as an anion channel and co-localizes with H⁺-ATPase	[54, 55]
AQP7	II	Adipose tissue	Mediates transmembrane glycerol transport and participates in triglyceride breakdown; its expression is altered in obesity and significantly upregulated in peripheral blood, and it may serve as a potential regulator of metabolic disorders	[69–71]
AQP8	I	Only in the pancreas and colon	The regulation of bile secretion within the bile canaliculi	[58, 59]
AQP9	II	Hepatocytes and leukocytes	Plays a pivotal role in the early acute response to endotoxin-induced shock through activation of the NF-κB signaling pathway	[72]
AQP10	II	Only in the small intestine of the human body	Human AQP10 mediates glycerol efflux; it provides an alternative glycerol efflux pathway when AQP7 function is impaired and can compensate for defects in AQP7-mediated glycerol efflux	[75]
AQP11	III	testes and thymus(highly), kidney, liver, and intestines(lowly)	Participating in the glycosylation process and facilitating the movement of Polycystin-1 from the endoplasmic reticulum to the plasma membrane	[82]
AQP12	III	only in pancreatic acinar cells	Contributing to the release of digestive enzymes and fluids within pancreatic cells	[81, 83]

*I* Orthodox Aquaporins, *II* Aquaglyceroporins, *III* Non-orthodox Aquaporins

## Physiological functions of aquaporins

The physiological functions of AQP family members adhere to a core paradigm: transport activity constitutes their central function, whereas signal-related regulation represents an extension of their functional repertoire. However, different AQP isoforms vary considerably in their relative contributions to these two dimensions. The execution of these functions is intimately linked to the tissue- and cell-specific distribution patterns of individual AQP isoforms. The orthodox AQPs, comprising AQP0, AQP1, AQP2, AQP4, AQP5, AQP6, and AQP8, are principally dedicated to transmembrane water flux, a role that underpins systemic fluid homeostasis [87]. Aquaglyceroporins (AQP3, AQP7, AQP9, and AQP10) exhibit dual permeability to water and small solutes such as glycerol and play a critical role in energy metabolism [88]. Non-orthodox AQPs (AQP11 and AQP12), through their organellar localization, facilitate water transport across intracellular membranes and help maintain organellar homeostasis. Beyond these canonical channel functions, AQPs are also involved in more complex biological processes, including cell proliferation, migration, and inflammatory responses, by modulating intracellular signaling cascades. These extended roles give rise to three interrelated functional dimensions: acting as metabolic hubs [89], serving as dual mediators of injury and repair [90, 91], and functioning as non-transport signaling nodes. These dimensions do not operate in isolation; instead, they are integrated into a regulatory network that underpins fundamental physiological processes such as fluid balance, metabolic regulation, and tissue repair. Moreover, they provide a molecular basis for the pathological mechanisms underlying disease onset and progression. Dysregulation of AQP function has thus emerged as a critical contributing factor in a spectrum of disorders, including fluid imbalance syndromes, metabolic diseases, and tumor development [92]. Notably, the AQP family exhibits considerable functional redundancy alongside pronounced inter-isoform divergence. A case in point is AQP10, which has undergone pseudogenization in several species, underscoring that the physiological significance of individual AQP isoforms cannot be assumed uniformly across the family [74].

### Metabolic center: regulation of transport of water and small nutrient molecules

**Fig. 1:**
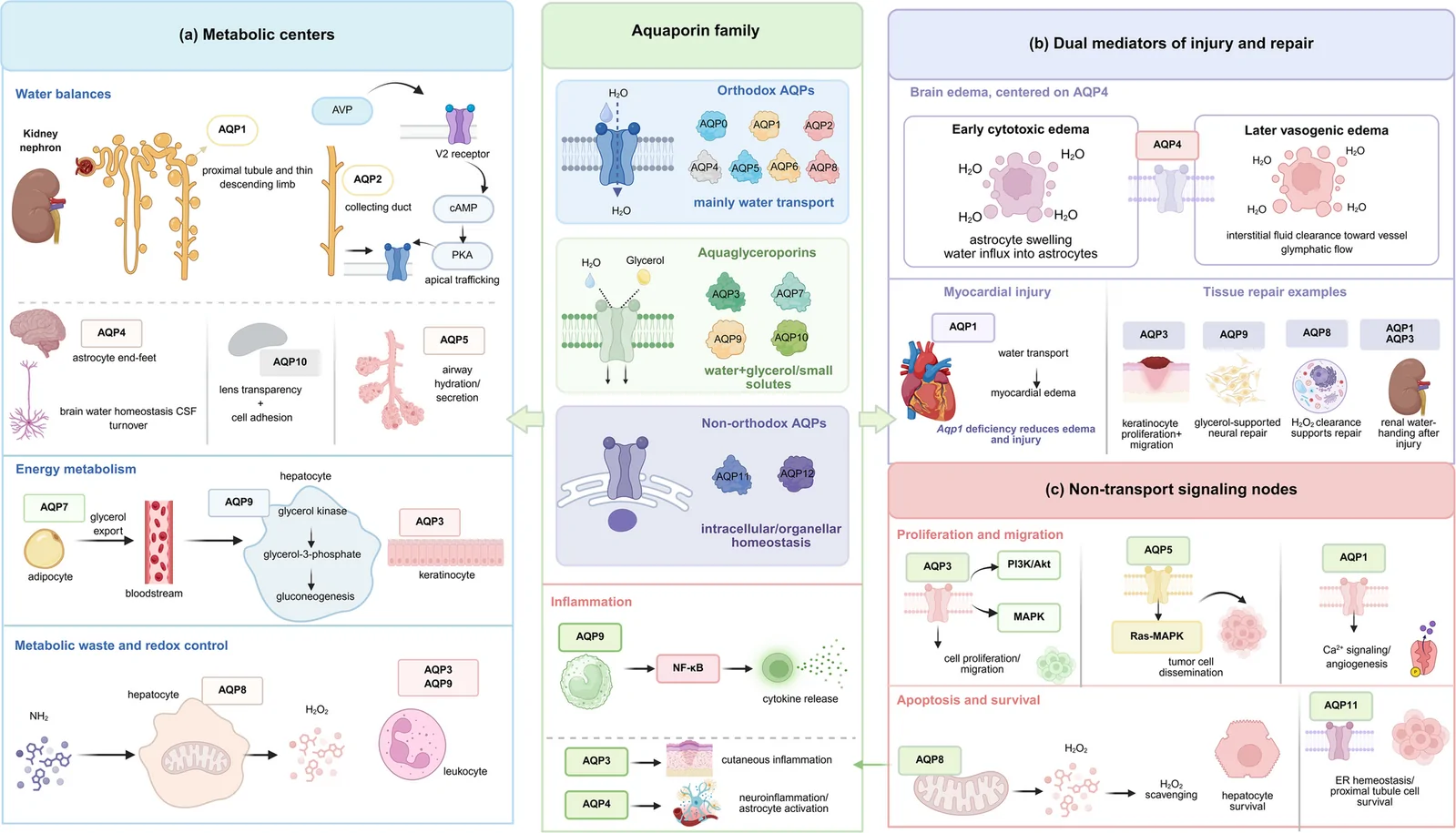
Physiological functions and pathophysiological roles of aquaporins. **a** Aquaporins regulate water homeostasis, energy metabolism, and metabolite/redox balance. The central schematic summarizes the major mammalian AQP subfamilies. **b** AQPs participate in tissue injury and repair, including edema regulation, tissue recovery, and inflammation. **c** AQPs modulate signaling pathways involved in cell proliferation, migration, apoptosis, and survival. Abbreviations:​ AVP, Arginine vasopressin; cAMP, Cyclic adenosine monophosphate; CSF, Cerebrospinal fluid; ER, Endoplasmic reticulum; H₂O₂, Hydrogen peroxide; NF-κB, Nuclear factor kappa B; PKA, Protein kinase A; ROS, Reactive oxygen species

As central metabolic regulators, AQPs are involved in key physiological processes, including fluid balance, energy metabolism, and lipid metabolism—by facilitating the transmembrane transport of small solutes such as water, glycerol, and urea [35, 60]. In doing so, they play an essential role in preserving systemic metabolic homeostasis [84] (Fig. 1a). Within this framework, aquaglyceroporins serve as the primary subtypes responsible for metabolic regulation, whereas orthodox AQPs provide the essential fluid equilibrium foundation that supports these metabolic functions [93].

#### Core regulation of water balance

Orthodox AQPs serve as essential regulators of body fluid balance, facilitating selective transmembrane water movement to preserve water-electrolyte homeostasis across most organ systems [5, 38]. Their physiological roles are shaped by subtype-specific tissue distribution and regulatory mechanisms [35]. Within the kidney, AQP1 is expressed in the proximal tubule and thin descending limb, where it contributes to constitutive water reabsorption, whereas AQP2 is expressed in collecting-duct principal cells and undergoes vasopressin-regulated trafficking to the apical membrane [94]. Recent studies have further identified TAZ–NR4A1 and AKAP–PKA-related mechanisms that regulate AQP2 abundance or activity in collecting-duct cells [11, 95]. Together, AQP1- and AQP2-dependent pathways contribute to urinary concentration. In the central nervous system, AQP4 is highly enriched at astrocytic perivascular end-feet, where α1-syntrophin-dependent anchoring contributes to its polarized distribution and bidirectional water movement between the brain and the vasculature [96]. The organization of AQP4 into orthogonal arrays is also influenced by the M1 and M23 isoforms [97]. In pathological conditions, disruption of AQP4 polarization or membrane trafficking can alter brain-water movement and glymphatic function [19, 98]. AQP0 represents the most abundant protein in lens fiber cell membranes, distinguished by its dual capability as a water channel and a cell adhesion molecule. This dual role is critical for preserving the compact organization and transparency of lens fibers; any functional disturbance can compromise lens osmotic stability and eventually lead to cataract formation [40]. In the respiratory tract, AQP5 is expressed in pulmonary epithelial cells and submucosal glandular structures. Recent human stem-cell-based studies have further characterized AQP5 expression in alveolar type I-like cells [14], whereas experimental studies in salivary glands have examined the developmental regulation of AQP5 [13]. These findings support a role for AQP5 in epithelial water handling, although its quantitative contribution to airway-surface-liquid regulation remains context-dependent.

#### Key mediators of energy metabolism

Aquaglyceroporins facilitate glycerol movement across cell membranes and thereby connect adipose lipolysis with hepatic glycerol utilization. During fasting in mice, AQP7-mediated glycerol efflux from adipose tissue facilitates the release of lipolysis-derived glycerol into the circulation [99]. In adipose tissue, for instance, AQP7 resides on the plasma membrane of both white and brown adipocytes. Upon triglyceride breakdown catalyzed by hormone-sensitive lipase, AQP7 releases the generated glycerol into the bloodstream, thus preventing intracellular glycerol accumulation that would otherwise cause feedback suppression of lipolysis [99]. Circulating glycerol is taken up by hepatocytes, where AQP9 provides a major route for glycerol entry and supports glycerol-derived gluconeogenesis in mice [100]. Additional experimental studies indicate that hepatocyte AQP8 and AQP9 jointly influence hepatic lipid and glucose metabolism, inflammatory signaling, and redox status [101]. In the skin, AQP3 is predominantly expressed in keratinocytes, where it transports glycerol from deeper epidermal layers toward the surface. This glycerol supply provides precursors for the synthesis of epidermal lipids, including ceramides, thereby promoting the formation of the stratum corneum lipid framework and maintaining skin barrier integrity [102, 103].

#### Regulation of excretion of metabolic wastes

At physiological pH 7.4, ammonia exists predominantly as ammonium ions (NH₄⁺), with an NH₄⁺/NH₃ ratio of approximately 99:1 (pKa = 9.25). The aqueous pore of AQPs is lined by hydrophobic residues and the conserved aromatic/arginine (ar/R) selectivity filter, whose narrow constriction (about 2.8 Å) imposes steric hindrance and electrostatic repulsion, thereby selectively permitting the passage of neutral small molecules such as water and NH₃ while excluding positively charged NH₄⁺ ions. Consequently, AQPs transport neutral NH₃ but are impermeable to NH₄⁺ [104]. However, AQPs are not the only pathways responsible for ammonia and urea movement across biological membranes. Rhesus family glycoproteins provide additional routes for ammonia transport. In the kidney, Rh B glycoprotein and Rh C glycoprotein are expressed in polarized epithelial membrane domains and participate in transepithelial ammonia handling, particularly in the distal nephron and collecting duct. Urea transport is mainly mediated by the dedicated urea transporter families UT-A and UT-B, which support rapid facilitated urea movement in the kidney and other tissues. These transport systems provide parallel and partly compensatory routes. Therefore, alterations in AQP expression or permeability are not necessarily predictive of whole-tissue ammonia or urea flux, which reflects the integrated contributions of AQPs, Rh glycoproteins, UT proteins, and other ion transport mechanisms [105, 106].

Selected AQP isoforms facilitate the transport of metabolic solutes, but their permeability profiles are isoform-, species-, and assay-dependent. AQP3 and AQP9 can transport glycerol and selected additional solutes. Human AQP8 facilitates ammonia and hydrogen peroxide transport but does not exhibit detectable urea permeability. Urea permeability has instead been reported for mouse AQP8 in selected heterologous expression systems. In parallel, this same subtype exerts an important function within mitochondria. Using the rat insulinoma-derived RINm5F cell line, Krüger et al. demonstrated that AQP8 facilitates hydrogen peroxide transport across mitochondrial and plasma membranes. By exporting H₂O₂ generated inside mitochondria to the cytoplasm, it helps cells clear oxidative stress products, thereby safeguarding mitochondrial integrity and preserving the normal metabolic function of insulin-secreting cells [107]. In the context of immune cells, both AQP3 and AQP9 participate in eliminating metabolic waste. A recent study by Garra et al. adopted a selective blocking strategy and demonstrated that suppressing AQP3 and AQP9 expression markedly impairs human leukocyte migration. This finding indicates that these two aquaglyceroporins not only manage the transmembrane movement of metabolites but also secure proper leukocyte movement and immune function by maintaining intracellular metabolic stability [17]. It is worth stressing that the functional specificity described above is not coincidental; rather, it stems from the precise subcellular localization of each subtype. In hepatocytes, AQP8 is found both at the plasma membrane and on mitochondrial membranes, allowing it to handle ammonia and hydrogen peroxide transport, respectively. Meanwhile, the presence of AQP3 and AQP9 on the leukocyte plasma membrane underlies their dual involvement in exporting metabolic waste and regulating cell migration. In summary, by virtue of their precise subcellular distribution and substrate selectivity, various AQP isoforms constitute a metabolite transport network spanning multiple organs and organelles, and they play an indispensable role in maintaining metabolic homeostasis.

As metabolic hubs, AQPs contribute to the maintenance of systemic metabolic homeostasis through three interconnected functional dimensions: (i) the fundamental regulation of fluid balance, (ii) the transport of small-molecule nutrients, and (iii) the facilitation of metabolic waste excretion. Their metabolic roles are characterized by both organ specificity and subtype cooperativity. The distinct tissue- and cell-specific distribution patterns of orthodox AQPs versus aquaglyceroporins establish an organ-preferential division of labor in metabolic regulation, while functional synergy among subtypes ensures the continuity and integration of metabolic processes. This precise alignment between cellular localization and physiological function represents a defining feature of AQP-mediated metabolic control.

### Dual mediation of injury repair: edema regulation and tissue repair

AQPs function as dual-effect mediators in the context of tissue injury and repair, exerting bidirectional regulatory influences on both the development and resolution of edema as well as on subsequent tissue regeneration [108]. Their specific contributions are modulated by factors such as the nature of the insult, the stage of pathological progression, and the distinct properties of individual subtypes. As pivotal regulators of post-injury homeostasis restoration, AQPs exert this dual functionality in a manner intrinsically dependent on the tissue- and cell-specific localization of each AQP subtype (Fig. 1b).

Within the central nervous system, AQP4 is recognized as a central regulator of cerebral edema. Its selective enrichment at astrocytic perivascular endfeet underpins an essential role in water movement across the brain [108]. Importantly, AQP4's influence on edema is not static but shifts according to edema subtype and the phase of the pathological process. In cytotoxic edema [109], for example, in cell swelling triggered by early cerebral ischemia or meningitis, water primarily flows from the vascular space into the brain parenchyma. In this setting, AQP4 facilitates rapid water entry into astrocytes, thereby worsening cell swelling. Using a mouse model of bacterial meningitis with AQP4 gene knockout, Papadopoulos and Verkman demonstrated that loss of AQP4 markedly reduces brain water accumulation and lowers mortality. These observations directly support a pro-edematous role for AQP4 during cytotoxic edema [110]. Conversely, during the resolution stage of vasogenic edema, when blood–brain barrier disruption causes extensive fluid leakage into the brain interstitium, AQP4 instead promotes water movement back from the brain parenchyma into the vasculature, thereby assisting edema absorption and clearance. A recent investigation by Zhu et al. demonstrated that glymphatic dysfunction after ischemic stroke is associated with persistent edema. As a key driver of interstitial fluid flow, AQP4 must function properly to resolve vasogenic edema [111]. Taken together, AQP4 acts as a double-edged sword in cerebral edema, exacerbating acute cellular swelling on the one hand while facilitating recovery-phase edema resolution on the other. The net outcome hinges on the pathological stage and the specific edema type. Compared with that of AQP4, the contribution of AQP1 and AQP9 to cerebral edema remains less well defined and may depend on species, cell type, and injury model.

AQP1 facilitates water transport in cardiomyocytes. Its selective distribution within myocardial tissue renders it​ a critical regulator of myocardial edema [112]. In mouse models of myocardial infarction, *Aqp1* deficiency reduces myocardial edema and limits infarct-associated tissue injury.

Following kidney injury, AQP1 and AQP3 play a role in the resulting water metabolism dysregulation. Because these AQPs have distinct distribution patterns within renal tissue, their dysregulated expression can exacerbate edema associated with acute kidney injury. Research has additionally demonstrated that under hypoxic conditions, AQP1 mRNA and protein levels are elevated both in vivo and in vitro [113]. This upregulation enhances water reabsorption in the proximal tubules, leading to reduced urine output in mice. However, whether this change mitigates edema remains to be determined.

During skin wound repair, AQP3 expression is upregulated in basal epidermal keratinocytes, where it promotes healing by facilitating glycerol transport and supporting cell proliferation and migration [66]. AQP9 expression has been reported to increase in reactive astrocytes after cerebral ischemic injury, particularly in rodent models. Because AQP9 can transport glycerol, it has been proposed to participate in astrocytic metabolic adaptation; however, direct evidence that AQP9 promotes neural tissue repair remains limited [114]. AQP8 can regulate H₂O₂ movement across intracellular membranes and thereby influence cellular redox balance. In rat insulinoma-derived RINm5F cells, AQP8 contributed to H₂O₂ transport across mitochondrial and plasma membranes [107]. In other experimental systems, stress-dependent changes in AQP8 permeability were associated with altered cell growth and survival [115]. Whether this mechanism directly promotes liver-tissue repair remains to be established. The reparative functions of different AQP isoforms are closely tailored to their specific tissue and cellular contexts.

As dual-role regulators of injury and repair, AQPs exhibit functional dynamics that are both disease stage-dependent and subtype-specific. During the early phase of injury, they predominantly promote edema formation and thereby exacerbate tissue damage; in contrast, during later stages, they contribute to edema resolution and tissue regeneration. The dominant regulatory subtypes differ across injured tissues: AQP4 plays a central role in the central nervous system, AQP1 in the myocardium, and AQP3 in the skin. This subtype specificity fundamentally reflects the precise tissue- and cell-specific localization of each AQP, while the observed stage-dependent behavior underscores the dynamic regulation of AQP function throughout the disease course.

### Non-transporting signal nodes: regulation of cell proliferation, migration and inflammation

Beyond their channel-mediated transport functions, AQPs can influence intracellular signaling pathways and cell behavior. In some settings, these effects may be partly independent of water or solute permeability, although transport-independent mechanisms have not been established for all AQP members or disease contexts [116–118]. Through modulation of these pathways, AQPs participate in key cellular processes such as proliferation, migration, apoptosis, and inflammatory responses (Fig. 1c). This signaling function, which may not rely on small-molecule transport, presents a significant expansion of AQP biology and serves as a central mechanism underlying their involvement in tumor progression and inflammatory diseases. These non-transport roles are closely linked to the tissue- and cell-specific localization of each AQP subtype.

AQP3 facilitates the proliferation and migration of both basal epidermal cells in the skin and various tumor cells through activation of signaling cascades such as PI3K/Akt and MAPK. Its distribution in the skin and in tumors makes it a key regulator of cellular dynamics in these contexts [119]. AQP5 enhances the proliferation and dissemination of lung cancer and glioma cells via engagement of the Ras-MAPK pathway, and its localization in pulmonary epithelial cells and glial cells confers tissue specificity to these oncogenic effects [120, 121]. AQP1 contributes to endothelial migration and pathological angiogenesis. Targeted AQP1 disruption reduced endothelial migration and tumor vascularization in experimental models [122, 123]. Whether these effects are entirely independent of AQP1-mediated water or solute transport remains unresolved. These signaling-mediated functions collectively highlight a non-transport dimension of AQP biology that is closely aligned with subtype-specific cellular distribution.

AQP3 modulates intracellular oxidative stress levels through hydrogen peroxide transport, thereby influencing apoptotic processes [124]. Its expression in metabolically active cells makes it a key regulator of apoptosis. AQP8 protects hepatocytes from apoptosis by scavenging hydrogen peroxide within mitochondria, with its mitochondrial localization being essential for hepatocyte survival [107]. AQP11 maintains endoplasmic reticulum homeostasis to inhibit apoptosis in renal proximal tubule cells, and its selective expression in this cell type underlies its specific role in renal cell survival [76]. The capacity to regulate apoptosis is fundamental to the maintenance of cellular viability and tissue integrity.

AQP9 facilitates the release of inflammatory cytokines in leukocytes via the NF-κB signaling pathway, thereby contributing to the regulation of endotoxin shock [72] and hepatic inflammation. Because AQP9 is expressed in both immune cells and hepatocytes, it acts as a central modulator of inflammatory processes. AQP3 contributes to cutaneous inflammatory responses by modulating signal transduction within skin cells [125]. AQP4 contributes to neuroinflammation by regulating astrocyte activation, positioning it as a key determinant in central nervous system inflammatory disorders [98]. The inflammatory regulatory functions of each subtype are closely aligned with their respective tissue- and cell-specific distribution patterns.

As non-transport signaling hubs, AQPs modulate key cellular processes, including proliferation, migration, apoptosis, and inflammation through the regulation of major signaling cascades such as MAPK, PI3K/Akt, and NF-κB [126, 127]. These signaling functions exhibit both subtype specificity and cell-type dependence, and in certain contexts, they operate synergistically with canonical transport activities to coordinate cellular physiological states. The tissue-restricted nature of these signaling roles is fundamentally dictated by the precise cellular localization of each AQP subtype, which in turn underpins their involvement in tumor biology and inflammatory diseases [128]. This mechanistic understanding provides a molecular basis for the development of targeted therapeutic strategies [32].

The physiological functions of AQPs are organized around three interconnected themes, reflecting a paradigm in which transporter activity serves as the foundation and non-transport functions represent an extension [5]. Functioning as metabolic hubs, they preserve systemic metabolic homeostasis through the transmembrane movement of small solutes such as water and glycerol [62, 63]; as dual-role mediators of injury and repair, they enable bidirectional regulation of edema and tissue regeneration in a disease stage-dependent manner [85, 91]; and acting as non-transport signaling nodes, they modulate cellular processes including proliferation, migration, and inflammation via the regulation of downstream signaling pathways. These three functional dimensions do not operate in isolation but are mutually interdependent and synergistic. For instance, the metabolic hub function supplies the material substrate necessary for signaling events, while signal regulation provides the cellular machinery that underpins tissue repair mechanisms [85, 91].

## Pathophysiology and functional disorders of aquaporins

### Molecular mechanisms of AQP dysfunction

The physiological roles of AQPs are contingent upon their proper expression, correct subcellular distribution, and structural integrity [5]. Consequently, genetic mutations, dysregulation of expression, and aberrant protein interactions constitute the three principal molecular mechanisms underlying AQP dysfunction [129–131]. Notably, these mechanisms may interact synergistically, thereby amplifying functional impairment [132]. Such pathological alterations ultimately disrupt the precise localization and functional execution of AQPs within specific tissues and cells, giving rise to a cascade of pathophysiological consequences [133].

Inherited mutations primarily lead to structural abnormalities or functional inactivation of AQPs, thereby compromising the physiological roles aligned with their tissue- and cell-specific distribution. Pathogenic AQP2 variants can impair protein folding, tetramer assembly, intracellular trafficking, apical membrane insertion, and channel function in principal cells of the renal collecting duct, thereby causing congenital nephrogenic diabetes insipidus [18, 134]. Pathogenic AQP0 variants can disrupt lens-fiber cell adhesion, membrane organization, water permeability, and lens transparency, thereby contributing to congenital cataract formation [40, 135]. Mutations in the V2 receptor cause a similar defect in collecting-duct water reabsorption, leading to nephrogenic diabetes insipidus [136, 137]. This finding is consistently observed in both human patients and knockout mouse models, underscoring the indispensable role of AQP2 in maintaining urinary concentration and systemic water balance. Such dysregulation causes the expression levels of specific AQP subtypes in particular tissues or cells to deviate from physiological requirements.

Acquired dysregulation of AQP expression is primarily driven by factors such as hormonal changes, signaling pathway alterations, and external stimuli, representing the predominant mechanism underlying AQP dysfunction in clinical disease. Such dysregulation causes the expression levels of specific AQP subtypes in particular tissues or cells to deviate from physiological requirements [138]. Chronic lithium exposure reduces AQP2 abundance and disrupts collecting-duct water reabsorption, thereby producing acquired nephrogenic diabetes insipidus [138, 139]. In experimental models, limiting lithium entry or attenuating endoplasmic-reticulum stress partially improved AQP2-related water-handling defects [140, 141]. In obesity, aberrant expression of AQP7 in adipose tissue [142] and AQP9 in the liver [143] contributes to the exacerbation of glucose and lipid metabolic disturbances. Upregulation of AQP3 and AQP5 in various tumor tissues enhances cancer cell proliferation and migration, while reduced AQP8 expression during liver fibrosis heightens oxidative stress in hepatocytes [144, 145]. Importantly, these expression anomalies are intimately linked to the tissue- and cell-specific distribution patterns characteristic of each AQP subtype.

Aberrant interactions between AQPs and their associated proteins lead to functional dysregulation, thereby compromising the physiological roles of these channels in specific tissues and cells. For example, disrupted interaction between AQP2 and Rab11 impairs recycling dynamics in renal collecting duct principal cells, leading to defective water reabsorption [146]. Similarly, altered binding between AQP4 and cytoskeletal components in astrocytes causes disassembly of orthogonal arrays of particles [96, 97], resulting in impaired water transport in the brain. In another instance, defective interaction between AQP11 and polycystin-1 leads to retention of polycystin-1 in the endoplasmic reticulum, contributing to renal cyst formation [147]. Notably, each of these pathological interactions specifically affects the function of a given AQP subtype within its specific cellular context.

The three principal molecular mechanisms responsible for AQP dysfunction are not mutually exclusive; instead, they interact and can trigger one another. For instance, genetic mutations may predispose cells to abnormal expression regulation, while external stimuli can concurrently induce both expression dysregulation and aberrant protein interactions [148]. Congenital mutations primarily underlie hereditary disorders by directly impairing the physiological functions adapted to subtype-specific tissue and cellular localization [129]. In contrast, acquired abnormalities, encompassing dysregulated expression and defective protein interactions are predominantly associated with non-hereditary conditions such as metabolic disorders, tumors, and inflammatory diseases. These acquired alterations cause functional impairment of AQP subtypes within specific cellular contexts and constitute primary targets for therapeutic strategies [149].

### Consequences of AQP dysfunction

AQPs, as critical regulators of systemic physiological homeostasis, when dysfunctional, give rise to four major pathophysiological consequences: disturbances in fluid balance [150], dysregulation of glucose and lipid metabolism [151], aberrant cellular behaviors [152], and inflammatory tissue injury [153]. These consequences do not occur in isolation but rather evolve through a "cascade effect" progressively advancing from molecular abnormalities to tissue- and organ-level pathological damage, ultimately contributing to disease initiation and progression. Importantly, each of these pathophysiological disturbances is closely linked to the tissue- and cell-specific distribution patterns characteristic of individual AQP subtypes.

Disorders of water homeostasis represent the most direct pathophysiological consequence of AQP dysfunction, primarily attributable to functional abnormalities in orthodox AQPs. For instance, impaired AQP2 function disrupts renal water reabsorption, culminating in diabetes insipidus [154]; dysfunction of AQP4 perturbs fluid homeostasis in the brain, leading to cerebral edema [155]; and abnormal AQP1 activity compromises aqueous humor and cerebrospinal fluid secretion, resulting in elevated intraocular and intracranial pressures [43, 155]. Each of these disturbances stems from the malfunction of specific AQP subtypes within organs critical for maintaining fluid balance.

Abnormalities in glucose and lipid metabolism are primarily driven by dysfunction of aquaglyceroporins and represent a core pathophysiological feature of metabolic disorders. For instance, dysregulation of AQP7 and AQP9 expression disrupts glycerol metabolism and hepatic gluconeogenesis, contributing to the development of obesity and diabetes [100, 156, 157]. AQP3 dysfunction impairs lipid synthesis in the skin, thereby compromising epidermal barrier integrity [158]. Similarly, aberrant AQP10 expression reduces glycerol absorption in the small intestine, further aggravating systemic metabolic imbalance [159]. These disturbances are directly attributable to the functional impairment of specific AQP subtypes within key metabolic organs.

Dysregulation of cellular behavior arises from aberrant signaling functions of AQPs independent of their transport activity, primarily manifesting as imbalances in proliferation, migration, and apoptosis. For instance, upregulation of AQP3 and AQP5 enhances tumor cell proliferation and migration, thereby facilitating oncogenesis and metastatic spread [144, 160, 161]; reduced AQP8 expression increases hepatocyte apoptosis, aggravating liver injury [162]; and elevated AQP1 levels promote abnormal endothelial cell proliferation, contributing to angiogenesis [163] and the progression of liver cirrhosis [164]. Importantly, these behavioral disturbances occur within specific cellular contexts and are closely associated with the distinct tissue and cellular localization of each AQP subtype.

Tissue inflammatory damage results from dysregulation of the inflammatory modulatory functions of AQPs, characterized by aberrant release of inflammatory cytokines and heightened oxidative stress. For example, upregulation of AQP9 promotes excessive secretion of inflammatory mediators by leukocytes, thereby aggravating endotoxic shock and hepatic inflammation [165]. Conversely, abnormal activation of AQP4 exacerbates astrocyte-mediated inflammatory responses, worsening central nervous system injury [166, 167]. Notably, each form of inflammatory damage occurs in tissues and organs that correspond precisely to the tissue distribution of the involved AQP subtype [5, 168]. The four major pathophysiological consequences of AQP dysfunction constitute a cascading pathological pathway that progresses from molecular abnormalities to cellular dysfunction, then to tissue- or organ-level damage, and ultimately to disease manifestation [35]. Furthermore, these consequences can reinforce one another: for instance, disruptions in glucose and lipid metabolism can intensify oxidative stress and inflammatory injury, while aberrant cellular behavior may promote tissue fibrosis and tumor development [169, 170]. The initiating event in this cascade is the dysfunction of specific AQP subtypes within defined cellular contexts, and the propagation of pathological effects relies on the precise tissue- and cell-specific distribution of these channels [33, 171]. This represents a fundamental mechanism … and provides critical targets for therapeutic intervention [32].

AQP dysfunction serves as a critical link between molecular alterations and the onset of disease [129]. Its three primary molecular mechanisms—gene mutations, dysregulated expression, and abnormal protein interactions—interact synergistically to trigger four major physiological disturbances: disrupted water metabolism, aberrant glucose and lipid metabolism, dysregulated cellular behavior, and tissue inflammatory damage [127]. Together, these form a progressive pathological cascade. Furthermore, the subtype specificity of AQP dysfunction determines the organ-specific manifestation of the disease, while the specificity of the functional themes involved dictates the resulting pathological phenotype [126].

It is instructive to note that, with the notable exceptions of AQP0, whose deficiency underlies congenital cataracts [172], AQP2, which is causally linked to nephrogenic diabetes insipidus [173], and AQP11, which has been implicated in renal cystogenesis [147], most AQP-knockout mouse models are characterized by remarkably mild physiological phenotypes. On the one hand, this phenomenon reflects functional redundancy among AQP family members, such as metabolic compensation between AQP7 and AQP10 [75]; on the other hand, it suggests that other compensatory water transport mechanisms may exist in the body. However, from the perspective of therapeutic development, the mild systemic effects of AQP knockout instead imply that AQP-targeted drugs may have low toxicity and limited side effects [174], creating favorable conditions for identifying highly specific AQP modulators.

## The role of AQPs in diseases

**Fig. 2:**
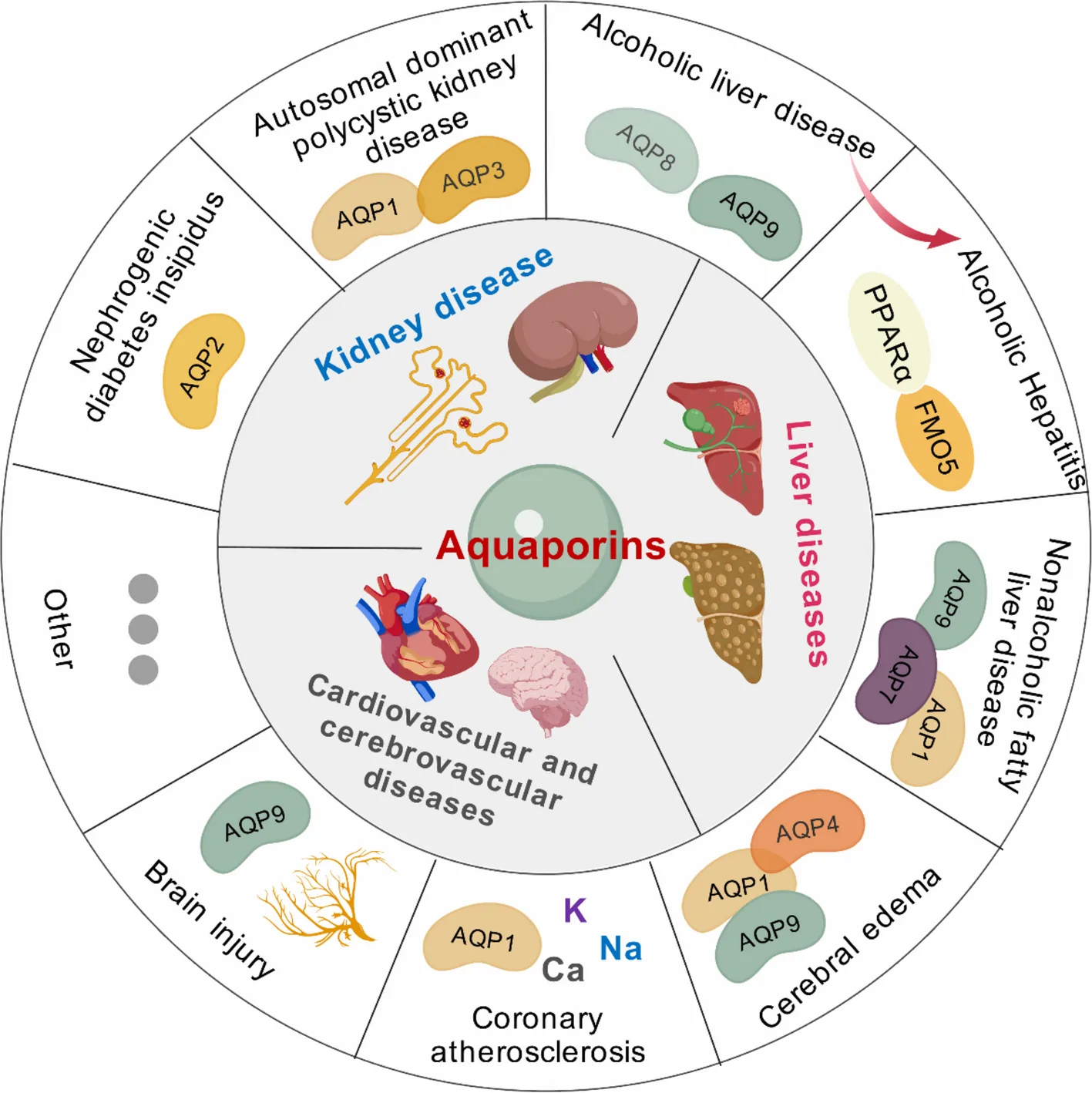
Roles of aquaporins in human disease pathogenesis. Different AQP isoforms participate in renal, hepatic, cardiovascular, and cerebrovascular disorders by regulating fluid homeostasis, metabolism, inflammatory responses, and cell behavior

To emphasize the functional consequences of primary-sequence divergence, disease-related evidence is reorganized here according to the three AQP subfamilies: orthodox aquaporins, aquaglyceroporins, and non-orthodox aquaporins [5, 175]. Within each subfamily, we discuss relevant organ systems and representative diseases [35]. This framework distinguishes the predominantly water-transport and edema-related roles of orthodox AQPs from the metabolic and redox functions of aquaglyceroporins and the organelle-associated functions of non-orthodox AQPs, as summarized in Fig. 2.

### Orthodox aquaporins

**Fig. 3:**
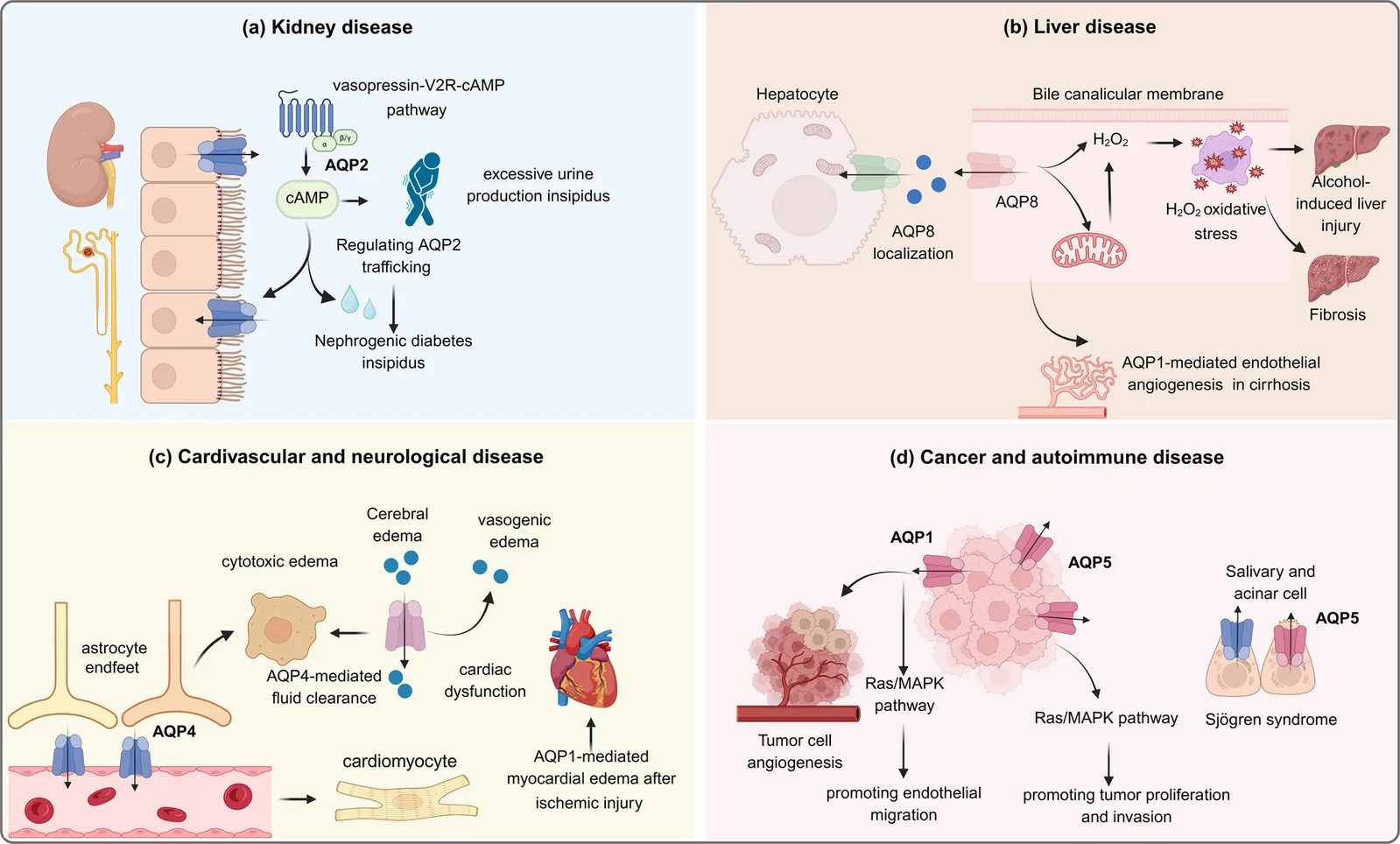
Roles of orthodox aquaporins in disease. **a** AQP2 dysfunction in kidney disease. **b** AQP8 and AQP1 involvement in liver injury and fibrosis. **c** AQP4 and AQP1 in neurological and cardiovascular injury. **d** AQP1 and AQP5 in tumor progression and Sjögren’s syndrome

Orthodox aquaporins are primarily associated with transmembrane water movement, epithelial secretion, tissue edema, and, in selected contexts, signaling and tumor progression [126, 148]. Current disease evidence is concentrated on AQP1, AQP2, AQP4, AQP5, and AQP8. The major disease-related roles of orthodox AQPs are summarized in Fig. 3.

#### Kidney diseases

Orthodox AQPs are central to renal water handling [176], and their dysfunction contributes particularly to nephrogenic diabetes insipidus (NDI), autosomal dominant polycystic kidney disease (ADPKD), and acute kidney injury [177–179]. The role of AQP2 dysfunction in NDI is summarized in Fig. 3a.

##### AQP2 and nephrogenic diabetes insipidus

Congenital NDI can result from AQP2 mutations, whereas acquired disease is commonly associated with reduced AQP2 abundance or defective trafficking [180]. Urinary AQP2 can provide information on renal responsiveness to vasopressin signaling [181]. Binding of arginine vasopressin to the V2 receptor elevates intracellular cAMP, promotes AQP2 insertion into the apical membrane, and limits endocytosis, thereby increasing collecting-duct water permeability and water reabsorption [182, 183]. Lithium exposure can reduce AQP2 expression and impair urine-concentrating capacity, thereby inducing or aggravating acquired NDI [140]. Preclinical approaches, including FMP-API-1 derivatives, vasopressin analogues, prostaglandin-receptor agonists, cGMP-phosphodiesterase inhibitors, and statins, have been proposed to enhance AQP2 expression or apical trafficking [95, 184]. However, the available human simvastatin study used urinary AQP2 as a physiological biomarker rather than evaluating clinical efficacy in NDI (NCT02523001). Therefore, dedicated dose-ranging trials with serum sodium and urine-concentrating endpoints are still required.

##### AQP1 and renal cystic or acute injury

AQP1 is expressed in the proximal tubule, the thin descending limb of the loop of Henle, and the descending vasa recta, where it supports water reabsorption and countercurrent urine concentration [185]. Experimental findings in cystic kidney disease are context-dependent: AQP1 deficiency has been linked to altered Wnt signaling and reduced cyst progression in some models, whereas embryonic kidney cultures have shown increased cystogenesis when osmotic water permeability is impaired [186, 187]. AQP1 loss also increases urine output in sepsis-associated acute kidney injury, indicating that its net effect depends on disease model, nephron segment, and stage.

#### Liver diseases

The liver expresses multiple AQP isoforms in hepatocytes, cholangiocytes, and vascular cells. Orthodox AQPs participate in bile secretion, redox control, angiogenesis, and hepatocellular survival, whereas aquaglyceroporins mainly regulate glycerol flux and energy metabolism [188, 189]. These roles are evident across alcoholic liver disease (ALD), non-alcoholic fatty liver disease (NAFLD), cirrhosis, and hepatocellular carcinoma (HCC). Among these processes, the roles of AQP8 and AQP1 in liver injury and fibrotic remodeling are summarized in Fig. 3b.

##### AQP8 in ALD

In hepatocytes, AQP8 is present in the smooth endoplasmic reticulum near glycogen particles and may facilitate rapid water movement during glycogen synthesis and breakdown [57]. At the canalicular membrane, AQP8 promotes bile flow and acetaldehyde excretion. Chronic alcohol exposure suppresses hepatic AQP8, leading to acetaldehyde accumulation, steatosis, inflammation, and fibrosis; conversely, restoring AQP8 reduces lipid deposition and immune-cell infiltration and downregulates *IL-6, CCL2, SREBP-1c*, and *SCD-1* [190–193]. Mitochondrial AQP8 is also permeable to H₂O₂ and contributes to the control of mitochondrial redox balance [115, 194]. By facilitating H₂O₂ efflux, AQP8 may limit oxidative injury and fibrogenic signaling.

##### AQP1 in NAFLD-associated cirrhosis

Angiogenesis contributes to fibrotic remodeling. Increased AQP1 expression in the neovasculature of cirrhotic liver enhances membrane dynamics, motility, and invasion of liver endothelial cells, suggesting that endothelial AQP1 may promote pathological angiogenesis and fibrosis [122, 195]. Nevertheless, therapeutic inhibition would require vascular selectivity to avoid disrupting physiological water transport.

##### AQP1, AQP5, and AQP8 in HCC

AQP5 expression is elevated in HCC tissues, and co-expression of AQP5 with the aquaglyceroporin AQP3 correlates with tumor stage, grade, metastatic potential, and prognosis [196]. AQP1 is detected predominantly in tumor microvessels rather than malignant hepatocytes and is associated with neovascularization, advanced disease, and adverse outcome [122, 197–200]. Mitochondrial AQP8 regulates metabolically generated H_2_O_2_ in human HepG2 cells and may influence apoptotic and necrotic cell death, indicating a context-dependent role in hepatocellular survival [92].

#### Cardiovascular and cerebrovascular diseases

In cerebral edema and ischemic myocardial injury [201], orthodox AQPs primarily regulate water flux, edema formation, and edema resolution, whereas aquaglyceroporins provide metabolic support during tissue repair, as shown in Fig. 3c.

##### AQP4 in cerebral edema

Cytotoxic edema involves intracellular water accumulation with a relatively intact blood–brain barrier, whereas vasogenic edema involves blood–brain barrier disruption and extracellular fluid accumulation [202, 203]. AQP4 is the principal astrocytic water channel involved in both processes, and its effect depends on edema type and disease stage [203, 204]. During cytotoxic edema, AQP4 facilitates water entry into astrocytes; AQP4 deletion reduces edema in models of water intoxication, cerebral ischemia, and bacterial meningitis and improves outcomes after transient middle cerebral artery occlusion [205, 206]. During vasogenic edema, however, AQP4 supports transcellular clearance of excess interstitial fluid toward cerebrospinal fluid and vascular compartments, and AQP4-deficient animals can develop more severe edema when the blood–brain barrier is disrupted [205, 206]. These apparently opposing effects are reconciled by the direction of the prevailing osmotic gradient, the relative contributions of cytotoxic and vasogenic edema, lesion location, and temporal stage [204]. Accordingly, therapeutic modulation of AQP4 should be phase- and edema-subtype-specific rather than uniformly inhibitory.

##### AQP1 in myocardial injury

AQP1 deletion in experimental myocardial infarction reduces myocardial edema and infarct size and improves cardiac function [207]. AQP1 has also been reported to exhibit a cGMP-regulated monovalent cation conductance, suggesting a potential link to cardiac excitability; however, the physiological and therapeutic relevance of this conductance remains to be further validated [208].

#### Nervous system diseases

AQP4 is the dominant disease-relevant orthodox AQP in the central nervous system. Its polarized localization at astrocytic end-feet and ependymal surfaces supports water balance, cerebrospinal-fluid circulation, blood–brain barrier function, and glymphatic clearance [108, 209–211]. Loss of expression or polarity therefore affects edema, neuroinflammation, protein clearance, and tissue repair, as shown in Fig. 3c.

##### AQP4 in alzheimer’s and Parkinson’s diseases

In Alzheimer’s disease, perivascular AQP4 supports cerebrospinal fluid/interstitial fluid exchange and facilitates elimination of β-amyloid and hyperphosphorylated tau. Loss of AQP4 polarity impairs glymphatic drainage, promotes neurotoxic-protein accumulation, and aggravates neuronal injury [212–216]. In Parkinson’s disease (PD), dopaminergic neurodegeneration and α-synuclein (α-SYN) aggregation provide the pathological context for AQP4 dysfunction [217]. In A53T model mice, blocking cerebral lymphatic drainage disrupts the exchange between cerebrospinal fluid and interstitial fluid, impairs AQP4 polarization in the substantia nigra, worsens α-SYN aggregation, damages dopaminergic neurons, and accelerates PD progression [218]. Additionally, AQP4 expression in astrocytes reduces α-SYN mRNA levels [219]. Beyond its role in α-SYN clearance, AQP4 also participates in regulating neuroinflammation and neurotrophic factors. In MPTP-induced PD models, AQP4-deficient astrocytes fail to upregulate transforming growth factor (TGF)-β1-a neuroprotective factor-indicating that AQP4 promotes TGF-β1 expression and thereby offers indirect neuroprotection [219]. Similarly, glial cell line-derived neurotrophic factor, which supports dopaminergic neuron survival, is downregulated in astrocytes lacking AQP4, suggesting an indirect protective effect of AQP4 on these neurons [220]. Moreover, in midbrain astrocytes of AQP4-knockout mice, the IKK/NF-κB pathway leads to increased TNF-α and IL-1β, confirming that AQP4 suppresses inflammatory pathways [221]. Given these regulatory mechanisms, AQP4 is strongly associated with PD and holds considerable therapeutic promise for the future.

##### AQP4 in neuromyelitis optica spectrum disorder (NMOSD)

AQP4 is the principal autoantigen in NMOSD. Patient-derived AQP4-IgG binds AQP4 on astrocytic end-feet and activates complement-dependent cytotoxicity, antibody-dependent cellular cytotoxicity, and AQP4 internalization, leading to astrocyte injury, blood–brain barrier disruption, inflammation, oligodendrocyte damage, and demyelination [222–225]. Competitive blockade with the non-pathogenic antibody aquaporumab reduces AQP4-IgG binding and tissue injury in cellular, ex vivo, and mouse models [226], supporting AQP4-directed intervention while underscoring the need for clinical validation.

#### Cancer

Abnormal expression of orthodox AQPs is associated with tumor-cell proliferation, migration, invasion, angiogenesis, and drug resistance [86]. Their effects vary by isoform, tumor lineage, and molecular context, as shown in Fig. 3c.

##### Breast cancer

AQP5 promotes triple-negative breast cancer growth, migration, and invasion and has been linked to EGFR/ERK and related oncogenic signaling [24]. AQP1 contributes to leptin-induced vasculogenic mimicry through the Ob-R/STAT3 axis and thereby supports perfusion-like channel formation and aggressive behavior [227]. These findings indicate distinct roles for epithelial AQP5 and vascular or tumor-associated AQP1.

##### Colorectal cancer

The role of AQP8 in colorectal cancer is far from straightforward, with accumulating evidence pointing to its dual nature. Zhang and colleagues observed that AQP8 transcript levels tend to be reduced in colorectal tumor tissues, and elevated expression of this gene is frequently linked to a more favorable clinical outcome. Functional assays conducted in vitro revealed that boosting AQP8 expression curbs tumor cell proliferation, migration, and invasion-a finding that was further supported by in vivo experiments, where AQP8 overexpression suppressed tumor growth in animal models. Mechanistically, AQP8 was identified as a direct downstream target of miR-92a, which drives the malignant behavior of colorectal cancer cells by negatively regulating AQP8 [228]. Subsequent work by Wu et al. offered additional insights, showing that enforced AQP8 expression markedly impairs the growth, colony-forming ability, and invasive potential of two colorectal cancer cell lines, SW480 and HT-29. From a signaling perspective, this suppressive effect can be largely ascribed to AQP8‑mediated inactivation of the PI3K/AKT axis and downregulation of protocadherin 7. Moreover, data from xenograft and metastasis models in nude mice further reinforce the view that AQP8 plays a critical role in restraining colorectal cancer progression and spread [31]. It is worth noting, however, that an earlier investigation reported high AQP8 expression in metastatic colon cancer specimens and a capacity to promote epithelial‑mesenchymal transition. Collectively, these seemingly disparate observations suggest that AQP8 may play a context-dependent, bifunctional role in colorectal cancer, with its precise effect largely determined by the tumor’s molecular background and the stage of disease progression.

##### Lung cancer

AQP1 is upregulated in non-small-cell lung cancer tissues, and its expression level is positively correlated with the abundance of neovascularization within tumors. Mechanistically, AQP1 promotes tumor angiogenesis by enhancing the motility of endothelial cells and facilitating the formation of lumen-like structures; conversely, inhibiting AQP1 can reduce microvessel density in lung cancer tissues, thereby slowing down tumor growth. A clinical study further indicated that overexpression of AQP1 is an independent risk factor for poor prognosis in patients with lung adenocarcinoma and is significantly associated with shorter progression-free survival [229]. AQP5 is increased in lung squamous-cell carcinoma and promotes epithelial-mesenchymal transition and invasive behavior through Ras/MAPK-related signaling [51].

##### AQP1 and tumor angiogenesis

AQP1 is enriched in vascular endothelium and promotes migration by supporting rapid local water flux at the leading edge of motile cells. Aqp1 deletion markedly reduced tumor vascularity, growth, and endothelial migration in an implanted-tumor model [122], and pharmacological inhibition of AQP1-dependent angiogenesis subsequently impaired melanoma growth in mice [123]. These experiments provide stronger causal evidence than immunohistochemical correlations alone. However, AQP1 is also required for physiological endothelial and epithelial water transport, so systemic inhibition may produce vascular or organ-specific toxicity; tumor-selective delivery and endothelial pharmacodynamic endpoints will be essential.

##### AQP5 modulates EMT via the Wnt/β-catenin signaling axis

Knockdown of *Aqp5* suppresses Wnt/β-catenin pathway activity, leading to reduced expression of EMT-related transcription factors and mesenchymal markers, which in turn dampens the migratory and invasive capabilities of colorectal cancer cells [230].With respect to chemoresistance, the link between AQP5 and 5-fluorouracil (5-FU) resistance has been systematically characterized. Stable silencing of AQP5 in HCT116 and HT29 colorectal cancer cell lines enhanced their chemosensitivity to 5-FU, as evidenced by stronger 5-FU-triggered apoptosis, inhibited tumor growth, and diminished in vivo resistance to 5-FU [230]. Mechanistically, the influence of AQP5 on 5-FU sensitivity is mediated through the Wnt/β-catenin pathway: AQP5 silencing dampens this pathway, thereby downregulating its downstream effector proteins. A critical rescue experiment showed that reintroducing a degradation-resistant β-catenin mutant, which lacks the GSK-3β phosphorylation site and thus accumulates persistently within cells, reversed the apoptosis induced by AQP5 silencing. This directly confirms that the Wnt/β-catenin cascade serves as the key downstream mediator of AQP5-modulated 5-FU sensitivity. Additional mechanistic insights reveal that AQP5 overexpression elevates p-NF-κB protein levels, activating NF-κB signaling to promote colorectal cancer cell proliferation and 5-FU resistance [29].

##### AQP5 in tumor progression and metastasis

AQP5 can promote proliferation, migration, epithelial-mesenchymal transition, and drug resistance through EGFR/ERK/p38-MAPK, Ras/MAPK, Wnt/β-catenin, and NF-κB-related pathways. In lung-cancer models, increased AQP5 enhanced proliferation and metastatic behavior, whereas reduced expression attenuated these phenotypes [51]. In colorectal cancer, AQP5 silencing decreases Wnt/β-catenin signaling and can increase sensitivity to 5-FU [29, 230]. Recent work also supports context-dependent roles of AQP5 in breast and gastrointestinal tumor cell states [24, 148]. Nevertheless, AQP5 is indispensable for normal exocrine secretion, creating a therapeutic trade-off between antitumor efficacy and xerostomia or ocular-surface toxicity.

#### Sjögren’s syndrome

##### AQP5 in exocrine-gland physiology and Sjögren's syndrome

AQP5 is concentrated at the apical membrane of salivary and lacrimal acinar cells, where it supports the transcellular water flux required for saliva and tear secretion [53]. Human studies in Sjögren's syndrome, however, have yielded inconsistent results. Steinfeld et al. reported redistribution of AQP5 from the apical membrane toward the basolateral membrane of acinar cells, consistent with defective secretion [231], whereas Beroukas et al. found no significant difference in AQP5 abundance or localization between patients and controls [232]. Differences in biopsy site, disease activity, fixation, antibody specificity, and quantitative definition of membrane localization may partly account for this discrepancy. Additional mechanisms include impaired muscarinic-receptor signaling, altered interactions with anchoring or secretory proteins, and autoimmunity: anti-AQP5 antibodies have been detected in patient sera and were associated with lower resting salivary flow, although their diagnostic sensitivity and specificity were only moderate [233, 234]. Thus, reduced secretion cannot be attributed solely to lower total AQP5 expression. A more plausible therapeutic objective is restoration of regulated apical trafficking and channel function in viable acinar cells, while recognizing that irreversible glandular destruction will limit the efficacy of AQP5-centered interventions, as shown in Fig. 3d.

#### Other orthodox AQP-associated disorders

AQP0 mutations disrupt lens-fiber water permeability and cell adhesion and are a well-established cause of congenital cataracts [40, 135, 172, 235]. By contrast, the direct contribution of AQP6 to human disease remains insufficiently defined, despite its distinctive acid-activated anion-channel properties. These differences illustrate that classification as an orthodox AQP does not imply uniform substrate selectivity or disease relevance.

### Aquaglyceroporins

**Fig. 4:**
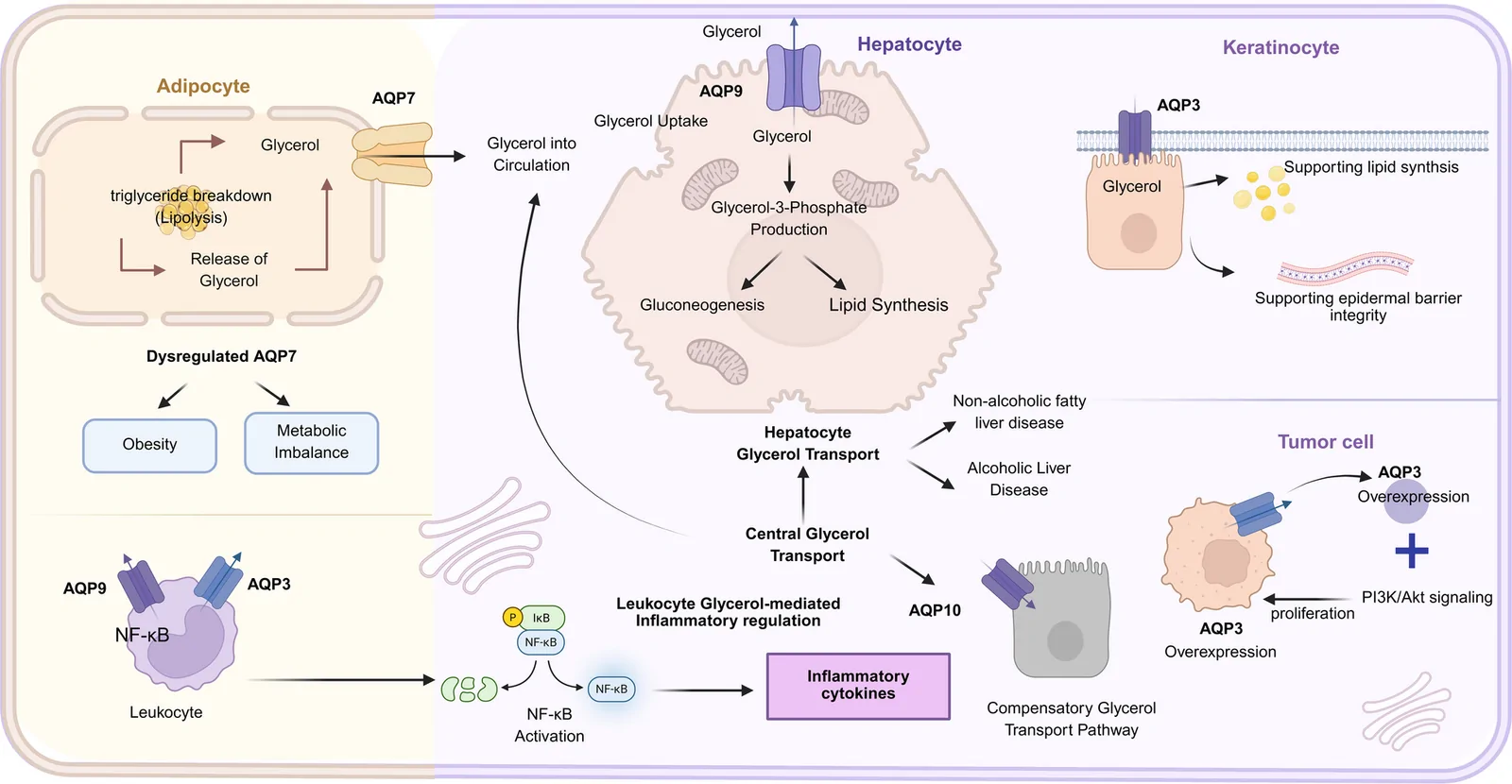
Roles of aquaglyceroporins in disease. Aquaglyceroporins regulate glycerol metabolism, inflammatory signaling, tumor progression, and metabolic homeostasis through the actions of AQP3, AQP7, AQP9, and AQP10. Abbreviations: AQP, aquaporin; NF-κB, nuclear factor kappa-light-chain-enhancer of activated B cells; PI3K, phosphoinositide 3-kinase; Akt, protein kinase B; NAFLD, non-alcoholic fatty liver; ALD, alcoholic liver disease

Aquaglyceroporins (AQP3, AQP7, AQP9, and AQP10) transport water and glycerol and, in some systems, H₂O₂, urea, or ammonia. Their disease roles cluster around altered energy metabolism, redox-sensitive signaling, immune-cell behavior, and tumor progression, as summarized in Fig. 4.

#### kidney diseases

##### AQP3 in autosomal dominant polycystic kidney disease

AQP3 can modulate diverse intracellular signaling pathways that regulate processes such as cell proliferation, apoptosis, and migration by facilitating the transport of glycerol and hydrogen peroxide across the cell membrane [236]. AQP3 is primarily located on the basolateral plasma membrane of the principal cells in renal collecting ducts and contributes to the regulation of urine concentration [144]. AQP3 may promote glycolysis by enhancing the transport of glycerol. Glycerol serves as a substrate for energy metabolism: it is phosphorylated by glycerol kinase to glycerol-3-phosphate, which is then converted to dihydroxyacetone phosphate (DHAP) and enters glycolysis. DHAP is a key glycolytic intermediate that contributes to ATP synthesis. Studies have shown that disrupted glucose metabolism is a hallmark of ADPKD [237]. The prevention of p-AMPK reduction in AQP3-MDCK cells during glucose deprivation suggests that glucose metabolism may be involved in the downregulation of p-AMPK. Consequently, inhibiting AQP3 could suppress glucose metabolism and slow ADPKD progression.

#### Liver diseases

##### AQP9 plays a role in the pathogenesis of alcoholic liver disease

AQP9 functions as the predominant facilitative diffusion channel governing glycerol influx into hepatocytes. During the initial 24 h of fasting, the rate of hepatic glycerol utilization is constrained by the AQP9-mediated uptake step, and when systemic glucose production is required, AQP9 operates at maximal capacity to optimize hepatic glycerol extraction [100]. Acute acetaldehyde-induced cell swelling has sometimes been attributed to AQP9, but the prevailing interpretation favors direct physicochemical disruption of the lipid bilayer rather than specific channel transport [238]. Alcohol-associated metabolic stress can disrupt insulin-dependent regulation of AQP7 and AQP9 [239]. Under physiological conditions, insulin suppresses adipose AQP7-mediated glycerol release and hepatic AQP9-mediated glycerol uptake, thereby limiting gluconeogenesis [240, 241].

##### AQP7 and AQP9 in NAFLD

AQP9 functions as a major glycerol channel in hepatocytes, where it participates in lipogenesis and gluconeogenesis, thereby exerting a regulatory influence on systemic energy homeostasis. The coordinated actions of AQP9 in the liver and AQP7 in adipose tissue are integral to the maintenance of adipose lipid storage, hepatic fat content, and systemic glucose equilibrium [142, 242, 243]. Nevertheless, the literature on AQP9 expression and function in NAFLD remains conspicuously discordant, with apparent contradictions likely attributable to differences in disease severity, experimental models, and metabolic milieus. On the one hand, several studies have documented elevated AQP9 expression in NAFLD patients and animal models, which facilitates hepatic glycerol uptake and gluconeogenic flux, thereby aggravating steatosis. On the other hand, an opposing body of evidence suggests that AQP9 downregulation may serve as a protective adaptive response, curtail the availability of triglyceride precursors and thus restrain pathological lipid accumulation. Furthermore, AQP9 ablation has been linked to exacerbated ER stress in fatty liver disease, with alterations in glycoprotein structure potentially triggering ER stress and reflecting hepatocellular injury [243, 244]. Of note, AQP9 deficiency in high-fat diet-fed mice has failed to uniformly reduce hepatic triglyceride content as predicted, and exhibits sexual dimorphism, highlighting the nuanced nature of its metabolic influence. Collectively, these observations caution against a simplistic interpretation of AQP9 as a straightforward therapeutic target for NAFLD. Although targeted modulation of AQP7 and AQP9 expression may, under selected conditions, attenuate hepatic steatosis and prevent the formation of large lipid droplets, the translational promise of such interventions requires rigorous validation through longitudinal studies designed with disease stage stratification and detailed subgroup analyses [244]. Thus, AQP7/AQP9 modulation requires disease-stage stratification rather than simple universal inhibition.

##### AQP3 in HCC

AQP3 is upregulated in HCC tissue, and its co-expression with AQP5 correlates with tumor stage, grade, metastatic potential, and prognosis [196]. Combined detection may therefore have biomarker value, although the independent contribution of AQP3 and its causal role in HCC progression require further mechanistic validation.

##### AQP10 and species-specific metabolic implications

Human AQP10 is expressed in the small intestine, adipose tissue, and liver, can compensate for AQP7-dependent glycerol efflux, and is reduced in obstructive cholestasis [73, 75, 245]. Because Aqp10 is a pseudogene in mice, conventional murine models cannot directly reproduce human AQP10 biology [74]. This species difference should be considered when translating aquaglyceroporin-based metabolic interventions.

#### Cerebrovascular diseases

##### AQP9 ischemic injury and repair

In the setting of ischemic injury, AQP9 expression is progressively upregulated in reactive astrocytes encircling the infarct core, with transcript and protein levels exhibiting a time-dependent increase; notably, this upregulation appears to be dissociated from the severity of the underlying brain insult [203]. Accumulating evidence indicates that AQP9 participates in astrocytic glucose and energy metabolism through its ability to facilitate transmembrane glycerol flux. Moreover, circulating insulin concentrations elevated pharmacologically or physiologically correlate with reduced AQP9 abundance in the cerebral parenchyma, suggesting a potential role for AQP9 in sustaining neuronal energy homeostasis through glycerol delivery [41]. To our knowledge, no published data is currently available regarding the involvement of the non-orthodox AQPs, AQP11 and AQP12, in the pathophysiology of cerebral edema.

#### Cancer

##### AQP3 in migration and proliferation

AQP3 couples glycerol and H₂O₂ transport to energy production, redox-sensitive signaling, and membrane protrusion. In colorectal carcinoma cells, AQP3 is required for epidermal-growth-factor-induced migration and is associated with adverse clinicopathological features [246]. Breast- and lung-cancer studies further show that the consequences of AQP3 silencing depend on molecular subtype, PI3K/AKT activity, and macrophage-derived cytokine signaling [247–249]. Accordingly, AQP3 is mechanistically plausible as a target, but its contribution should be confirmed using transport-deficient mutants or selective inhibitors rather than inferred solely from knockdown or expression data.

Collectively, aquaglyceroporins connect substrate transport with metabolic and redox signaling. Their context-dependent effects and compensatory relationships, particularly among AQP7, AQP9, and AQP10, argue for therapeutic strategies that are specific to isoform, tissue, disease stage, and species.

### Non-orthodox aquaporins

**Fig. 5:**
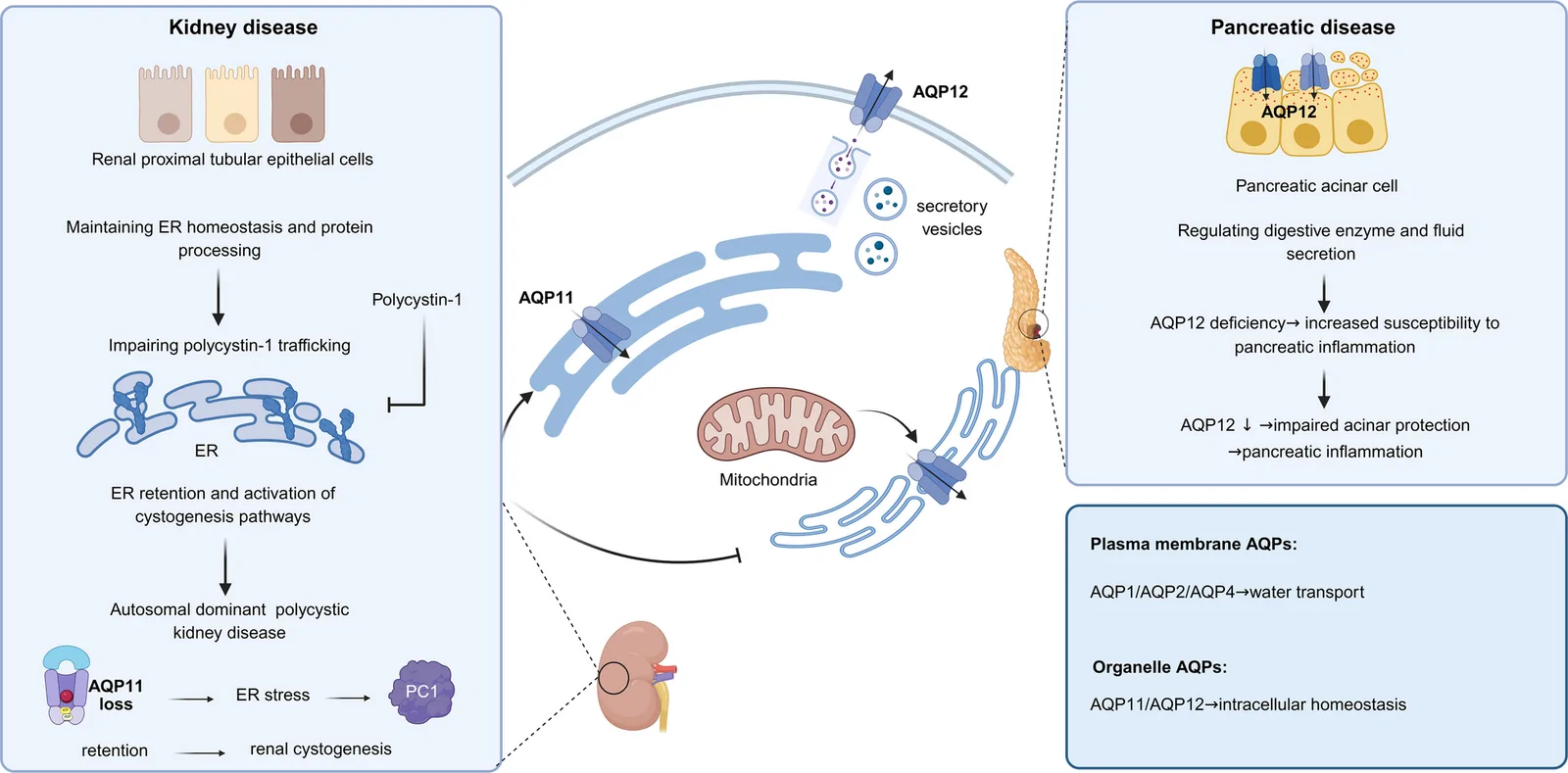
Roles of non-orthodox aquaporins in disease. AQP11 is predominantly localized to the endoplasmic reticulum and contributes to organelle homeostasis and protein processing. AQP11 deficiency impairs polycystin-1 maturation and trafficking, thereby promoting renal cyst formation. AQP12 is mainly expressed in pancreatic acinar cells and participates in secretory processes; its deficiency increases susceptibility to pancreatic injury and acute pancreatitis

The non-orthodox aquaporins AQP11 and AQP12 are structurally divergent and are mainly localized to intracellular organelles [76, 250]. Their disease associations are therefore more closely related to organellar homeostasis, protein processing, and organ-specific injury than to bulk transepithelial water transport, as summarized in Fig. 5.

#### Kidney diseases

##### AQP11 in renal cystogenesis

AQP11 is expressed in renal proximal tubules and contributes to endoplasmic-reticulum homeostasis and protein glycosylation. AQP11 facilitates the processing and trafficking of polycystin-1 from the endoplasmic reticulum to the plasma membrane; AQP11 dysfunction causes polycystin-1 retention in the endoplasmic reticulum and promotes renal cyst formation [82]. This mechanism directly links an organelle-localized AQP to the pathogenesis of polycystic kidney disease and distinguishes AQP11 from AQP1 and AQP3, which act primarily at the plasma membrane.

#### Pancreatic disease and current evidence gaps

AQP12 is enriched in pancreatic acinar cells and has been investigated mainly in experimental pancreatitis. *Aqp12*-deficient mice display greater pancreatic injury after induction of pancreatitis despite relatively mild changes under basal conditions [81, 83]. Beyond this context, evidence remains sparse. No convincing direct role for AQP11 or AQP12 has yet been established in ALD, NAFLD, HCC, cerebral edema, or the major cancers discussed above. The limited evidence base precludes firm therapeutic conclusions and highlights the need for organelle-resolved assays, human-relevant models, and isoform-specific modulators.

In summary, reorganizing disease evidence by AQP subfamily reveals a clear functional hierarchy: orthodox AQPs dominate disorders of water balance, secretion, and edema; aquaglyceroporins connect glycerol and H₂O₂ transport to metabolism, inflammation, and tumor behavior; and non-orthodox AQPs regulate intracellular organelle homeostasis but remain comparatively underexplored. This distinction provides a more mechanistically informative basis for evaluating AQP-targeted therapies.

## AQP modulators

AQPs serve as critical mediators governing water transport, metabolic processes, and signal transduction pathways within living organisms. Dysfunction of these channels is intimately linked to the initiation and progression of a broad spectrum of human diseases. Consequently, AQP-targeted modulators, capable of either activating or inhibiting channel activity-have emerged as promising therapeutic candidates and represent a focal point in contemporary biomedical research. To date, identified AQP modulators encompass a diverse array of agents, ranging from small-molecule compounds and biological therapeutics to natural products. Most AQP modulators remain at the preclinical stage, having been validated only in vitro or in animal studies [251–253]. Although certain compounds such as propofol [254], atorvastatin [255], and curcumin [256] are clinically approved (as anesthetics, lipid-lowering agents, and dietary supplements, respectively), their approved indications do not target AQPs, and they have not yet been translated into clinical practice as AQP-targeted therapies. Moreover, tailored modulatory strategies have been developed to target distinct AQP subtypes, thereby establishing a framework for subtype-selective intervention, as shown in Table 2.

**Table 2:** AQPs modulators

Types	Representative drugs	Targeting AQP subtypes	Mechanism of action	Main application directions	Highest phase
Inhibitor	TGN-020	AQP4	(a) Selectively targeting the extracellular domain including the water pore; (b) impeding transmembrane water flux in astrocytes, mitigating fluid accumulation in brain interstitial space	Cerebral edema (ischemic stroke, traumatic brain injury)	Preclinical [19, 109, 251, 257]
Inhibitor	ORI-TRN-002	AQP4	(a) Engineering via structural refinement of TGN-020; (b) comparable mechanism with improved aqueous solubility, tissue penetrability, and reduced cytotoxicity	Cerebral edema (optimized candidate for clinical advancement)	Preclinical [252]
Inhibitor	Propofol	AQP4	Modulating transcription factors to suppress AQP4 expression and diminishing membrane localization of functional AQP4 protein	Cerebral ischemia–reperfusion injury; clinically approved as anesthetic (non-AQP indication)	preclinical [254]
Inhibitor	anti-AQP3 monoclonal antibody	AQP3	(a) Specific binding to the extracellular epitope of AQP3 and blockade of its interaction with glycerol and H₂O₂; (b) suppression of keratinocyte hyperproliferation and T cell-driven inflammation	Psoriasis (imiquimod-induced model)	Preclinical [258, 259]
Inhibitor	Copper sulfate (CuSO₄)	AQP3	Interacts with metal-binding domains of AQP3, inducing conformational changes that impede transmembrane transport of glycerol and H₂O₂	Tumor intervention (reduces glycerol uptake, mitochondrial ATP production, and cancer cell proliferation)	Preclinical [260–262]
Inhibitor	propofol	AQP3	(a) Modulating PI3K/Akt signaling, leading to transcriptional suppression of AQP3 expression in lung cancer cells; (b) reducing membrane localization of functional AQP3	Lung cancer (inhibiting invasion and metastasis of cancer cells)	Preclinical [263]
Inhibitor	BAY-876	AQP1	specifically inhibits GLUT1, altering intracellular glucose metabolism, which indirectly modulates membrane localization and activity of AQP1	Tumor angiogenesis, cardiovascular disease	Preclinical [264, 265]
Inhibitor	Z433927330	AQP7 (also partial AQP3)	Potent inhibition of murine AQP7-mediated water flux (IC₅₀≈0.2 μM) and glycerol permeation; originally characterized as a partial AQP3 inhibitor (IC₅₀≈0.7–0.9 μM)	Limited translation due to suboptimal isoform selectivity and pharmacokinetic profiles	Preclinical [266, 267]
Inhibitor	Z225008686 (in silico predicted)	AQP7	Computational prediction of AQP7 inhibitory activity; biological relevance remains experimentally unsubstantiated	Not yet validated experimentally	In silico [266, 267]
Inhibitor	RG100204	AQP9	(a) Obstructing AQP9-mediated passage of water, glycerol, and H₂O₂; (b) suppressing NF-κB activation; (c) downregulating the NLRP3 inflammasome and caspase-1; (d) activating Nrf2	Sepsis (multi-organ dysfunction), severe acute pancreatitis (SAP), ARDS, cardiac/renal/hepatic injury	Preclinical [268–271]
Activator	FMP-API-1	AQP2	Disrupting AKAP–PKA interactions increase PKA-dependent phosphorylation of AQP2 at Ser256, which promotes apical accumulation in collecting duct principal cells	Nephrogenic diabetes insipidus (impaired AQP2 trafficking)	Preclinical [95, 272]
Activator	FMP-API-1/27	AQP2	Analog with greater activity; disrupts AKAP–PKA interactions to promote AQP2 apical accumulation via PKA-dependent Ser256 phosphorylation	Nephrogenic diabetes insipidus (impaired AQP2 trafficking)	Preclinical [272]
Activator	Statins (Atorvastatin, Simvastatin)	AQP2	(a) Enhancing AQP2 apical membrane accumulation via inhibition of the mevalonate pathway; (b) vasopressin-independent strategy	Nephrogenic diabetes insipidus; clinically approved as lipid-lowering agents (non-AQP indication); clinical trials: NCT02523001 (observational), NCT02967653 (Phase 2 RCT)	Clinical (repurposed) [255, 273]
Natural Product Activator	Curcumin	AQP8	(a) Activating Nrf2 signaling, enhancing transcriptional expression of AQP8 in hepatocytes; (b) augmenting H₂O₂ transport and clearance capacity	Hepatic steatosis, oxidative liver injury, intestinal inflammation; clinically approved as dietary supplement (non-AQP indication)	Preclinical [256]
Natural Product Modulator	AQP9 knockdown (lentivirus-mediated RNAi)	AQP9	Knockdown reduces hepatocyte glycerol uptake and lipid accumulation, alleviating hepatic steatosis	Non-alcoholic fatty liver disease	Preclinical [274]
Natural Product Modulator	Resveratrol (dietary polyphenol)	AQP1, AQP3, AQP7	activating the SIRT1 signaling pathway, regulating expression balance of multiple AQP subtypes (AQP1, AQP3, AQP7)	Glucose/lipid metabolic disturbances, intestinal barrier integrity, gut health	Preclinical [275]

### AQP inhibitors

AQP inhibitors are currently the most widely studied type of modulators, mainly targeting core subtypes such as AQP1, AQP3, and AQP4, focusing on the intervention of diseases like edema, tumors, and inflammation.

#### AQP1 inhibitor

Among compounds that indirectly modulate AQP1, BAY-876 (N-(4-methoxybenzyl)-N-(5-nitro-1,3-thiazol-2-yl) urea), a compound frequently used in preclinical studies, exerts its effect through an indirect mechanism. Rather than directly targeting AQP1, BAY-876 specifically inhibits the glucose transporter GLUT1 [264]. By altering the intracellular glucose metabolic profile, it indirectly modulates the membrane localization and functional activity of AQP1. This indirect effect suppresses water transport in tumor vascular endothelial cells and impedes neovascularization, suggesting therapeutic potential in oncology and cardiovascular disease [265].

#### AQP3 inhibitor

Currently, the well-established AQP3 inhibitors in research mainly fall into two categories: biological agents and small molecule compounds.

As a representative biological agent, the anti-AQP3 monoclonal antibody specifically recognizes and binds to an extracellular epitope of AQP3, effectively blocking its interaction with substrates including glycerol and hydrogen peroxide [259]. This blockade inhibits AQP3-mediated transport activity, thereby suppressing keratinocyte hyperproliferation and T cell-driven inflammatory responses. Such interventions demonstrate pronounced therapeutic efficacy in the imiquimod-induced psoriasis model [258].

With regard to small-molecule inhibitors, cupric sulfate (CuSO₄) interacts with the metal-binding domain of AQP3 [260], thereby inducing conformational changes that impede the transmembrane transport of glycerol and hydrogen peroxide [261]. This mechanism provides a foundation for subsequent investigations into tumor intervention. From the standpoint of tumor regulatory pathways, glycerol serves as a crucial substrate for energy metabolism in cancer cells. The inhibition of AQP3-mediated glycerol uptake directly reduces the influx of glycerol into tumor cells, leading to diminished synthesis of glycerol-3-phosphate. Consequently, mitochondrial ATP production becomes less efficient, reducing the energy supply required for the rapid proliferation of malignant cells [262].

Another small-molecule inhibitor, propofol, modulates the PI3K/Akt signaling pathway, leading to transcriptional suppression of AQP3 expression in lung cancer cells. This results in reduced membrane localization of functional AQP3 protein, thereby impairing the invasive and metastatic capabilities of lung cancer cells [263].

#### AQP4 inhibitor

Regarding AQP4 inhibitors, the small-molecule compound TGN-020 (4-[4-chlorophenyl) methyl]−1-[4-nitrophenyl) sulfonyl] piperidine) is a prominent representative [109]. This agent selectively targets the extracellular domain of the AQP4 channel, including the water pore structure [19]. By impeding transmembrane water fluxes in astrocytes, it reduces excessive fluid accumulation in the brain interstitial space [109]. Consequently, this mechanism alleviates cerebral edema resulting from conditions such as ischemic stroke and traumatic brain injury [251, 257].

Based on this work, the novel inhibitor ORI-TRN-002 was developed through structural refinement. It shares a similar mode of action with TGN-020 but has substantially improved aqueous solubility and tissue penetration, along with reduced cytotoxicity [252]. These improved properties make it a more viable candidate for clinical development.

Propofol (2,6-diisopropylphenol) modulates the activity of specific transcription factors, thereby suppressing AQP4 expression in astrocytes and reducing membrane localization of functional AQP4. This mechanism attenuates cellular edema and tissue damage caused by cerebral ischemia–reperfusion injury, underscoring its therapeutic potential for neurological disorders [254].

#### AQP7 inhibitor

At present, the discovery and development of isoform-selective small-molecule inhibitors targeting AQP7 remain in their infancy. Among the limited number of reported AQP7 modulators, Z433927330 is the most extensively studied compound to date. This molecule was originally characterized as an AQP3 inhibitor (IC₅₀ ≈ 0.7–0.9 μM) [266]. However, subsequent studies showed that it also potently inhibits murine AQP7-mediated water flux (IC₅₀ ≈ 0.2 μM) and reduces glycerol permeation across erythrocyte membranes. Stopped-flow light scattering was the primary biophysical assay used for functional characterization. Furthermore, computational approaches have generated predictions of additional lead candidates, such as Z225008686, but their biological relevance remains to be experimentally substantiated [266, 267]. Collectively, the current generation of AQP7 inhibitors suffers from suboptimal isoform selectivity, modest binding affinity, and unfavorable pharmacokinetic profiles, with none having advanced to clinical development. This developmental bottleneck has, in turn, constrained the translational exploration of AQP7 as a viable therapeutic target.

#### AQP9 inhibitor

RG100204, a selective inhibitor of AQP9, confers protection against septic injury by interfering with the transport functions of the channel [268]. By specifically obstructing AQP9-mediated passage of water, glycerol, and H₂O₂, this compound disrupts key pathological processes in sepsis. Experimental evidence demonstrates that RG100204 effectively suppresses mouse AQP9-dependent H₂O₂ flux in vitro, with a half-maximal inhibitory concentration (IC₅₀) of approximately 1.2 × 10⁻⁷ M. Furthermore, treatment with this inhibitor markedly attenuates the generation of both superoxide anions and nitric oxide in FaO hepatoma cells challenged with LPS [72, 268]. In a murine model of polymicrobial sepsis induced by cecal ligation and puncture, the pharmacological blockade of AQP9 by RG100204 dampens pro-inflammatory signaling cascades. This is evidenced by diminished activation of the NF-κB pathway-characterized by reduced phosphorylation of IKKα/β and IκBα alongside decreased nuclear translocation of the p65 subunit-as well as downregulated expression of the NLRP3 inflammasome and reduced cleavage of caspase-1 within cardiac and renal tissues [268]. The compound also limits pulmonary inflammation, as reflected by a significant decrease in myeloperoxidase activity within lung tissue, suggesting restricted neutrophil recruitment and activation [269, 270]. Chen et al. showed that AQP9 inhibitor RG100204 alleviated pancreatic and lung injury in severe acute pancreatitis (SAP) models via NF-κB/NLRP3 suppression and Nrf2 activation, highlighting AQP9 inhibition as a potential strategy for SAP and its systemic complications, including acute respiratory distress syndrome [271]. Collectively, these interconnected mechanisms contribute to the alleviation of sepsis-induced multiple organ dysfunction, encompassing impairments in cardiac performance as well as injury to the kidneys and liver.

### AQP activators

AQP activators mainly focus on AQP2, which is primarily used in the treatment of nephrogenic diabetes insipidus, and they mostly function by activating signaling pathways or upregulating expression.

AQP2 trafficking provides the clearest rationale for indirect AQP modulation. Studies of hereditary nephrogenic diabetes insipidus established that several AQP2 mutants retain water-channel function but are trapped in the endoplasmic reticulum or Golgi apparatus; a dominant-negative effect can occur when a misrouted mutant forms heterotetramers with wild-type AQP2 and prevents apical delivery of the wild-type channel [276]. This work supports trafficking rescue rather than nonspecific pore activation. FMP-API-1 disrupts AKAP-PKA interactions, increases PKA-dependent phosphorylation of AQP2 at Ser256, and promotes apical accumulation in collecting-duct principal cells [95]. Its analog FMP-API-1/27 shows greater activity in preclinical systems [272], but neither compound has entered human efficacy testing. Furthermore, statins can enhance apical membrane accumulation of AQP2 through inhibition of the mevalonate pathway, providing a vasopressin-independent strategy that has shown benefit in cellular and animal models of impaired AQP2 trafficking [273].

### New regulators

Natural products and bioactive dietary constituents represent an emerging class of AQP modulators. These compounds act mainly by modulating AQP expression. Curcumin is an illustrative example: it activates the Nrf2 signaling pathway, thereby enhancing AQP8 expression in hepatocytes. This upregulation augments the hydrogen peroxide transport and clearance capacity of AQP8, mitigating oxidative injury to liver cells [256]. This mechanism may therefore help me improve hepatic steatosis and intestinal inflammation. Cai et al. employed lentivirus-mediated RNA interference to knock down hepatic AQP9 expression in a high-fat diet-induced rat model of NAFLD, demonstrating that *Aqp9* knockdown reduces hepatocyte glycerol uptake and lipid accumulation, thereby alleviating hepatic steatosis [274]. Additionally, dietary polyphenols such as resveratrol serve as effective modulators of multiple AQP isoforms. Resveratrol activates the SIRT1 signaling pathway [277], and regulates the expression balance of multiple AQPs subtypes (such as AQP1, AQP3 and AQP7). This regulatory action contributes to the amelioration of glucose and lipid metabolic disturbances, restoration of intestinal barrier integrity, and subsequent preservation of overall gut health [275].

Clinical translation remains limited. No direct AQP pore inhibitor, activator, or isoform-specific biologic has yet established efficacy in a registered therapeutic trial. Human evidence is restricted mainly to repurposing or biomarker studies: NCT02523001 was a completed observational study of simvastatin and urinary AQP2 in hypercholesterolemic adults, not an interventional trial in nephrogenic diabetes insipidus, whereas NCT03349008 evaluated glycyrrhizin derivatives plus entecavir in chronic hepatitis B and did not test AQP9 modulation [86, 227, 228]. By contrast, the strongest mechanistic programs remain preclinical, including AQP4 inhibitors for acute cytotoxic edema, an anti-AQP3 antibody for inflammatory injury, selective AQP3/AQP7 small molecules, and the AQP9 inhibitor RG100204 in sepsis and pancreatitis models [271]. Future clinical development should prespecify direct target engagement, isoform selectivity, disease phase, pharmacodynamic AQP endpoints, adverse effects expected from physiological channel function, registry identifier, recruitment status, and clinically meaningful efficacy outcomes [32, 149, 278].

### Translational barriers in aquaporin-targeted therapeutics

Despite promising preclinical findings, the clinical translation of AQP-targeted therapies remains limited by four interrelated challenges. First, many reported small-molecule inhibitors exhibit insufficient affinity or isoform selectivity, whereas mercurial compounds are intrinsically toxic [169]. Second, the narrow and highly conserved architecture of AQP pores complicates conventional structure-based drug discovery [279]. Third, marked species differences reduce the predictive value of standard animal models. These include pseudogenization of *Aqp10* in mice, species-dependent substrate selectivity of AQP8, and the absence of constitutive AQP9 expression in healthy human astrocytes [39, 280]. For example, mouse AQP8 has been reported to facilitate urea transport in heterologous expression systems, whereas human AQP8 shows no detectable urea permeability [39]. Fourth, pharmacological conclusions are frequently derived from compounds with unrelated primary targets, antibodies of uncertain specificity [281], or changes in total AQP expression without direct assessment of membrane localization, target engagement, or substrate permeability. Overcoming these limitations will require rigorous target-engagement assays, transport-deficient controls, structurally distinct chemical scaffolds, human-relevant cellular or organoid models, and disease models that accurately reproduce the relevant pathological stage [282]. Collectively, these challenges help explain why no direct AQP modulator has yet progressed to confirmatory clinical development [98].

## Conclusions and perspectives

This review indicates that AQPs are multifunctional membrane proteins that integrate water and solute transport with metabolic regulation, redox signaling, tissue repair, and organ-level homeostasis. Their pathophysiological roles are highly context-dependent and cannot be inferred from expression abundance alone; isoform identity, subcellular localization, post-translational modifications, interacting partners, and the tissue microenvironment collectively determine their functional outcomes. This complexity explains both the broad involvement of AQPs in human disease and the persistent difficulty of translating AQP biology into clinical therapies.

Several limitations constrain current knowledge and translation. The narrow, highly conserved AQP pore architecture makes selective pharmacological targeting difficult, and most available inhibitors lack isoform specificity or sufficient blood–brain barrier penetration [283]. Functional redundancy and species differences further limit the predictive value of conventional animal models. Moreover, many reported AQP modulators have not been rigorously demonstrated to engage the intended target, alter membrane localization or transport activity, and produce AQP-dependent rescue effects [284]. These issues highlight the need for stricter mechanistic validation and more cautious extrapolation from preclinical data to human disease.

Future research should move from broad descriptions of AQP involvement toward mechanism-defined, context-dependent intervention. We propose four priorities. First, the cell-type-specific and organelle-specific functions of non-canonical AQPs, particularly AQP11 and AQP12, should be clarified [76]. Second, high-affinity, isoform-selective modulators should be developed through structure-based design, DNA-encoded libraries, and functional screening [32]. Third, AQP biomarkers should be validated in prospective clinical cohorts with clear analytical and clinical performance criteria [285]. Fourth, clinically testable protocols should be designed for AQP-driven pathologies such as cerebral edema, NAFLD, and metastatic disease, ideally using human-relevant models such as organoids and organ-on-a-chip systems. Humanized or conditional animal models will also be important for reducing species-dependent misinterpretation.

We believe that the next decade will shift AQP therapeutics from nonspecific pore blockade toward precise regulation of localization, trafficking, and context-specific signaling. If supported by rigorous target engagement and clinically anchored biomarkers, such strategies could transform AQPs from challenging membrane proteins into actionable therapeutic nodes. The field is already moving from "channel biology" toward "systems pathophysiology" and this integrated perspective is most likely to deliver meaningful advances in neuroprotection, metabolic regulation, and cancer therapy.

## Data Availability

Not applicable.

## Abbreviations

AQPs: Aquaporins
Ras-MAPK: Ras-mitogen-activated protein kinase
NF-κB signaling pathway: Nuclear factor-kappa B signaling pathway
PC-1: Polycystin-1
ADPKD: Autosomal dominant polycystic kidney disease
NDI: Nephrogenic diabetes insipidus
cAMP: Cyclic adenosine monophosphate
cGMP: Cyclic guanosine monophosphate
FMP-API-1: 3,3'-Diamino-4,4'-dihydroxydiphenylmethane
ALD: Alcoholic liver disease
ECM: Extracellular matrix
NAFLD: Nonalcoholic fatty liver disease
HCC: Hepatocellular carcinoma
NASH: Non-alcoholic steatohepatitis
p-AMPK: Phosphorylated AMP-activated protein kinase
PI3K/Akt: Phosphatidylinositol 3-kinase/Protein Kinase B
mTOR: Mammalian target of rapamycin
MAPK/ERK: Mitogen-activated protein kinase/Extracellular signal-regulated kinase
